# PAR1 activation induces rapid changes in glutamate uptake and astrocyte morphology

**DOI:** 10.1038/srep43606

**Published:** 2017-03-03

**Authors:** Amanda M. Sweeney, Kelsey E. Fleming, John P. McCauley, Marvin F. Rodriguez, Elliot T. Martin, Alioscka A. Sousa, Richard D. Leapman, Annalisa Scimemi

**Affiliations:** 1 SUNY Albany, Dept. Biology, 1400 Washington Avenue, Albany NY 12222, USA; 2 SUNY Oneonta, Dept. Computer Science, 108 Ravine Parkway, Oneonta NY 13820, USA; 3 National Institute of Biomedical Imaging and Bioengineering, National Institutes of Health, 9000 Rockville Pike, Bethesda MD 20852, USA

## Abstract

The G-protein coupled, protease-activated receptor 1 (PAR1) is a membrane protein expressed in astrocytes. Fine astrocytic processes are in tight contact with neurons and blood vessels and shape excitatory synaptic transmission due to their abundant expression of glutamate transporters. PAR1 is proteolytically-activated by bloodstream serine proteases also involved in the formation of blood clots. PAR1 activation has been suggested to play a key role in pathological states like thrombosis, hemostasis and inflammation. What remains unclear is whether PAR1 activation also regulates glutamate uptake in astrocytes and how this shapes excitatory synaptic transmission among neurons. Here we show that, in the mouse hippocampus, PAR1 activation induces a rapid structural re-organization of the neuropil surrounding glutamatergic synapses, which is associated with faster clearance of synaptically-released glutamate from the extracellular space. This effect can be recapitulated using realistic 3D Monte Carlo reaction-diffusion simulations, based on axial scanning transmission electron microscopy (STEM) tomography reconstructions of excitatory synapses. The faster glutamate clearance induced by PAR1 activation leads to short- and long-term changes in excitatory synaptic transmission. Together, these findings identify PAR1 as an important regulator of glutamatergic signaling in the hippocampus and a possible target molecule to limit brain damage during hemorrhagic stroke.

The G-protein coupled receptor PAR1 is a serine protease mostly expressed in brain astrocytes^1^,^2^. PAR1 is activated by bloodstream serine proteases like thrombin, the main effector molecule in the blood coagulation cascade, and plasmin, an enzyme involved in the proteolysis of fibrin blood clots^3^. Thrombin and plasmin activate PAR1 by cleaving its extracellular N-terminal domain, revealing a tethered agonist for PAR1^4^,^5^. Despite being irreversible, PAR1 activation remains effective for a limited time, due to rapid PAR1 internalization and lysosomal degradation^6^. *In vitro*, synthetic peptides that mimic the aminoacid sequence of the endogenous tethered ligand allow to reversibly and selectively activate PAR1 without affecting other potential targets of thrombin and plasmin^3^. Once activated, PAR1 interacts with proteins of the Gq/11, Gi/o, and G12/13 families, regulating signal transduction pathways leading to mobilization of intracellular calcium^7^,^8^,^9^. Consistent with these findings, in mouse hippocampal *stratum radiatum*, PAR1 activation evokes intracellular calcium rise in astrocytes, not neurons^10^.

Astrocytes are the most abundant non-neuronal cells in the brain. In the hippocampus, a brain region that plays a critical role in memory formation and generation of temporal lobe seizures^11^, fine astrocytic processes extend towards excitatory synapses^12^. They form physical barriers to neurotransmitter diffusion and terminate excitatory synaptic transmission by removing the neurotransmitter glutamate from the extracellular space through the activity of glutamate transporters^13^. Glutamate uptake is stoichiometrically coupled with the movement of Na^+^, H^+^ and K^+^ across the membrane, along the direction determined by their electrochemical gradient^14^,^15^,^16^,^17^. In acute brain slices, this stoichiometric and synaptically-activated transporter current (STC) can be monitored using whole-cell patch-clamp recordings from astrocytes^18^. The time course of the STC provides a temporally-distorted view of the lifetime of glutamate at astrocytic membranes^18^,^19^,^20^. Its time course depends on the spatial organization of astrocytic processes and synapses and on the density of expression of glutamate transporters (i.e. the astrocyte uptake capacity). Accordingly, STCs become faster during development, when the glutamate uptake capacity increases through developmental regulation of glutamate transporter expression^13^. The brain neuropil undergoes constant remodeling in physiological^21^ and pathological conditions^22^ and these phenomena can shape excitatory transmission in the brain. Whether PAR1 activation is involved in this remodeling is not known.

Here we show that, in area CA1 of the mouse hippocampus, PAR1 activation induces rapid and complex re-organization of the local neuropil surrounding excitatory synapses, which is associated with faster glutamate clearance, reduced glutamate receptor activation and impaired long-term potentiation (LTP). Together, these findings identify PAR1 as a powerful regulator of excitatory synaptic function in the brain and a potential molecular target to limit brain damage during pathological conditions associated with damage of the brain vasculature like traumatic brain injury, epilepsy and hemorrhagic stroke.

## Results

### PAR1 activation speeds clearance of synaptically-released glutamate from the hippocampal neuropil

To test whether PAR1 regulates the lifetime of synaptically-released glutamate at astrocyte membranes, we performed whole-cell patch-clamp recordings from hippocampal astrocytes in CA1 *stratum radiatum*, in response to Schaffer collateral stimulation. The recorded current consisted of a large, fast-rising inward component decaying in tens of milliseconds (i.e. the STC), and a slow-rising, sustained inward component decaying with a much slower time course of several seconds^18^ (Supp. Fig. 1A). The slow current is a sustained K^+^-current, which follows the time course of K^+^ re-equilibration in the extracellular space^18^. In a series of control experiments we confirmed that: (1) STCs are mediated by glutamate transporters because they are blocked by the broad-spectrum, non-substrate, competitive glutamate transporter antagonist TBOA (50 μM)^18^,^20^ (Supp. Fig. 1A); (2) the sustained and TBOA-insensitive K^+^-current was only partially blocked by BaCl_2_ (200 μM), a broad-spectrum blocker of K_ir_ channels abundantly expressed in glial cells of the retina^23^, olfactory bulb^24^ and somato-sensory cortex^25^ (normalized I_k_ amplitude in TBOA + BaCl_2_ versus TBOA: 0.52 ± 0.11 (n = 7), **p = 5.4e-3; data not shown). This effect might be due to the presence of Ba^2+^-insensitive K^+^ channels in hippocampal astrocytes^26^. We did not use BaCl_2_ in the rest of the experiments not only because it did not block the sustained K^+^ current, but also because it could change the dynamics of synaptic transmission by permeating through voltage-gated Ca^2+^-channels (substituting for Ca^2+^, though much less effectively, in triggering neurotransmitter release^27^). The kinetics of the STC are indistinguishable from those of the facilitated portion of STCs (i.e. the fSTC), isolated by interleaving single and paired stimuli to Schaffer collaterals^20^,^28^,^29^ (amp STC_TBOA sub_ 51.6 ± 16.8 pA, fSTC 54.3 ± 15.8 pA *p* = 0.54; 20–80% rise STC_TBOA sub_ 2.2 ± 0.4 ms, fSTC 1.8 ± 0.2 ms *p* = 0.36; τ_fast_ STC_TBOA sub_ 5.5 ± 0.7 ms, fSTC 5.9 ± 0.7 ms *p* = 0.70; τ_slow_ STC_TBOA sub_ 12.8 ± 0.7 ms, fSTC 11.2 ± 2.6 ms *p* = 0.57; <t>_TBOA sub_ 10.0 ± 1.9 ms, fSTC 9.4 ± 1.3 ms (n = 4) *p* = 0.84; Supp. Fig. 1B–F). Therefore, TBOA-subtracted STCs or fSTCs can be used interchangeably to estimate glutamate clearance. The amplitude and kinetics of fSTCs remain stable over a period of 30–40 min, the typical duration of our astrocyte whole-cell patch-clamp recordings (amp fSTC_Ctrl_ 49.0 ± 16.8 pA, fSTC_time_ 46.2 ± 15.6 pA *p* = 0.26; 20–80% rise fSTC_Ctrl_ 1.6 ± 0.1 ms, fSTC_time_ 1.8 ± 0.2 ms *p* = 0.09; τ_fast_ fSTC_Ctrl_ 5.7 ± 0.7 ms, fSTC_time_ 5.0 ± 0.4 ms *p* = 0.42; τ_slow_ fSTC_Ctrl_ 12.1 ± 2.3 ms, fSTC_time_ 15.0 ± 4.3 ms *p* = 0.32; <t>fSTC_Ctrl_ 8.3 ± 1.2 ms, fSTC_time_ 9.0 ± 1.1 ms (n = 4) *p* = 0.14; Supp. Fig. 1G–I). Their kinetics are not altered by the mGluRI antagonists LY367385 (50 μM) and MPEP (10 μM; 20–80% rise fSTC_Ctrl_ 1.8 ± 0.3 ms, fSTC_mGluRI_ 2.5 ± 0.5 ms *p* = 0.07; τ_fast_ fSTC_Ctrl_ 5.2 ± 0.9 ms, fSTC_mGluRI_ 5.4 ± 1.4 ms *p* = 0.88; τ_slow_ fSTC_Ctrl_ 12.7 ± 2.7 ms, fSTC_mGluRI_ 12.0 ± 1.7 ms *p* = 0.82 (n = 9)), in agreement with recent work indicating that mGluRI do not affect the glutamate transport process^30^ (Supp. Fig. 2). Therefore, we analyzed fSTCs recorded in the absence of BaCl_2_ and mGluRI antagonists to derive information on glutamate clearance.

**Figure 1:**
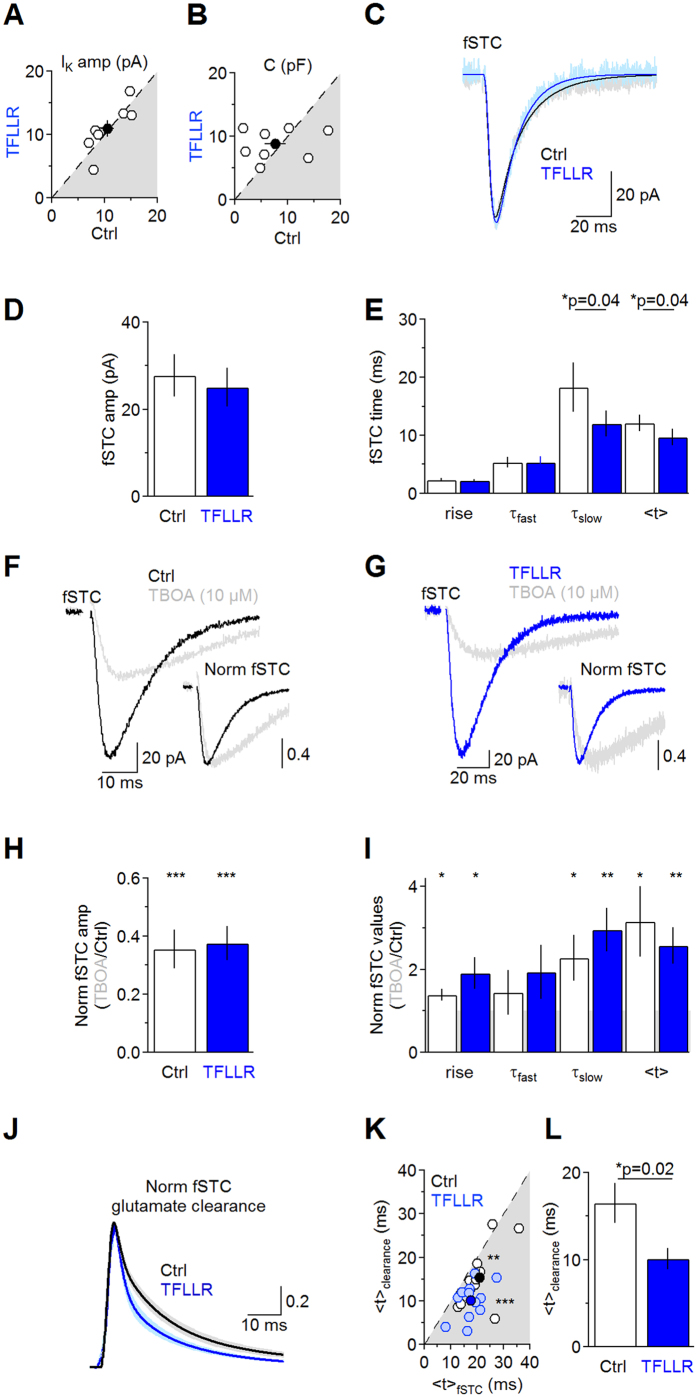
PAR1 activation speeds transporter currents and glutamate clearance from astrocytes. (**A**) In-cell comparison of the amplitude of the sustained K^+^-current in control (10.6 ± 1.2 pA) and TFLLR (30 μM; 10.9 ± 1.3 pA (n = 7), *p* = 0.64). TFLLR does not change the amplitude of the K^+^ -current. (**B**) In-cell comparison of astrocyte capacitance in control (7.7 ± 2.0 pF) and TFLLR (8.8 ± 0.9 pF (n = 7), *p* = 0.62). TFLLR does not change the astrocyte capacitance. (**C**) fSTCs in control (*gray*) and TFLLR (*light blue*). Averages of 20 consecutive fSTCs. Thick lines represent multi-exponential fits of the fSTCs before (*black*) and after TFLLR (*blue*). (**D**) Summary graph: TFLLR does not change the fSTC amplitude (Ctrl: 27.7 ± 4.9 pA; TFLLR: 25.0 ± 4.4 pA (n = 12), *p* = 0.40). (**E**) Summary graph: TFLLR speeds the fSTC slow decay (τ_slow_; Ctrl: fSTC 20–80% rise 2.3 ± 0.3 ms, τ_fast_ 5.3 ± 0.9 ms, τ_slow_ 18.3 ± 4.3 ms, <t> 12.1 ± 1.4 ms; TFLLR: fSTC 20–80% rise 2.3 ± 0.2 ms *p = *0.62, τ_fast_ 5.3 ± 1.0 ms *p = *0.94, τ_slow_ 12.0 ± 2.3 ms *p = 0.04, <t> 9.7 ± 1.4 ms (n = 12) *p = 0.04). **(F)** fSTCs in control (*black)* and TBOA (10 μM; *gray*). Average of 20 consecutive fSTCs. Insets: peak-normalized fSTCs. **(G)** As in (F), in TFLLR. (**H**) Summary graph: TBOA (10 μM) reduces the fSTC amplitude similarly in control (0.36 ± 0.07 (n = 10) ***p = 2.7e-6) and TFLLR (0.36 ± 0.06 (n = 10) ***p = 1.5e-6; Ctrl vs TFLLR *p = *0.92). (**I**) Summary graph: TBOA prolongs the fSTC rise and slow decay in control (fSTC Norm 20–80% rise 1.4 ± 0.1 *p = 0.02, Norm τ_fast_ 1.4 ± 0.5 *p = *0.43, Norm τ_slow_ 2.2 ± 0.6 *p = 0.04, Norm <t > 3.1 ± 0.8 (n = 10) *p = 0.03) and TFLLR (fSTC Norm 20–80% rise 1.9 ± 0.4 *p = 0.04, Norm τ_fast_ 1.9 ± 0.6 *p = *0.18, Norm τ_slow_ 2.9 ± 0.5 **p = 0.005, Norm <t> 2.6 ± 0.4 (n = 10) **p = 0.006). The effects of TBOA are similar in both conditions (Norm 20–80% rise *p* = 0.23, Norm τ_fast_ *p* = 0.56, Norm τ_slow_ *p* = 0.39, Norm <t> *p* = 0.56). **(J)** Derived clearance of synaptically-released glutamate in control (*black*) and TFLLR (*blue*). **(K)** The clearance centroid is faster than the fSTC centroid, in control (<t>_fSTC_ 23.3 ± 2.0 ms, <t> _clearance_ 16.5 ± 2.2 ms (n = 13) **p = 0.002) and TFLLR (<t>_fSTC_ 20.6 ± 1.8 ms, <t>_clearance_ 10.1 ± 1.2 ms (n = 12) ***p = 6.7e-4). **(L)** Summary graph: the centroid of glutamate clearance derived from fSTCs is faster in control (16.5 ± 2.2 ms (n = 13)) than TFLLR (10.1 ± 1.2 ms (n = 12) *p = 0.02).

TFLLR is a synthetic 5-aa peptide with an aminoacid sequence that mimics the sequence of the tethered ligand of PAR1^4^,^5^,^6^,^10^,^31^ (Fig. 1). TFLLR (30 μM), applied for 30 min, did not change the amplitude of the sustained K^+^ current (Ctrl 10.6 ± 1.2 pA, TFLLR 10.9 ± 1.3 pA (n = 7) *p* = 0.64; Fig. 1A), whole-cell capacitance (Ctrl 7.7 ± 2.0 pF, TFLLR 8.8 ± 0.9 pF (n = 7) *p* = 0.62; Fig. 1B), fSTC amplitude (Ctrl 27.7 ± 4.9 pA, TFLLR 25.0 ± 4.4 pA (n = 12) *p = *0.40; Fig. 1C,D), 20–80% rise (Ctrl 2.3 ± 0.3 ms, TFLLR 2.3 ± 0.2 ms (n = 12) *p = *0.62; Fig. 1E) and fast decay (Ctrl 5.3 ± 0.9 ms, TFLLR 5.3 ± 1.0 ms (n = 12) *p = *0.94; Fig. 1E). In contrast, TFLLR significantly reduced the slow component of the fSTC decay (Ctrl 18.3 ± 4.3 ms, TFLLR 12.0 ± 2.3 ms (n = 12) *p = 0.04), leading to a change of its centroid (<t>)^20^,^28^ (Ctrl 12.1 ± 1.4 ms, TFLLR 9.7 ± 1.4 ms (n = 12) *p = 0.02; Fig. 1E). Such a small effect on the fSTC might mask profound changes in glutamate clearance^18^.

We estimated the time course of glutamate clearance by using a deconvolution analysis of fSTCs^20^,^28^. Briefly, the waveform of the fSTC can be thought of as a mathematical transformation (i.e. a convolution) of glutamate clearance (an event that lasts a few milliseconds) and a distorting function (i.e. the filter). In our experimental conditions, the filter behaves as a linear system which is unaffected by changes in glutamate uptake capacity^20^,^28^,^29^. To derive the filter using the deconvolution analysis, we need to record fSTCs before and after applying a low, sub-saturating concentration of TBOA^20^,^28^. The goal of this manipulation is to block a significant fraction – not all – transporters, to *prolong* the time course of the fSTC without completely abolishing it^20^,^28^. When the glutamate uptake capacity of astrocytes is low, as it happens with TBOA (10 μM), the fSTC decay reflects more closely the time course of glutamate clearance (not the filter), which can be approximated by an instantaneously-rising function decaying with the same time course of the fSTC^20^,^28^. Deconvolving the filter derived in TBOA (10 μM) from the fSTC in control (when the uptake capacity is intact) allows deriving the time course of glutamate clearance in control conditions. TBOA (10 μM) induced a similar reduction of the fSTC amplitude in control (0.36 ± 0.07 (n = 10), ***p = 2.7e-6) and TFLLR (0.36 ± 0.06 (n = 10), ***p = 1.5e-6; Ctrl vs TFLLR *p = *0.92; Fig. 1F–H). TBOA (10 μM) did not affect the fast component of the fSTC decay, but prolonged its rise, slow decay and centroid, in control and TFLLR (fSTC Norm 20–80% rise Ctrl 1.4 ± 0.1, TFLLR 1.9 ± 0.4, *p* = 0.23; Norm τ_fast_ Ctrl 1.4 ± 0.5, TFLLR 1.9 ± 0.6, *p = *0.56; Norm τ_slow_ Ctrl 2.2 ± 0.6, TFLLR 2.9 ± 0.5, *p* = 0.39; Norm <t> Ctrl 3.1 ± 0.8 (n = 10), TFLLR 2.6 ± 0.4 (n = 10), *p* = 0.56; Fig. 1I). As expected^20^,^28^, the time course of glutamate clearance was significantly faster than the time course of the fSTC in control (<t>_fSTC_ 23.3 ± 2.0 ms, fSTC <t>_clearance_ 16.5 ± 2.2 ms (n = 13) **p = 0.002) and TFLLR (<t> _fSTC_ 20.6 ± 1.8 ms, fSTC <t>_clearance_ 10.1 ± 1.2 ms (n = 12) ***p = 6.7e-4; Fig. 1J,K). Notably, TFLLR sped the time course of glutamate clearance from astrocytes (<t>_fSTC_ Ctrl 16.5 ± 2.2 ms (n = 13), TFLLR 10.1 ± 1.2 ms (n = 12) *p = 0.02; Fig. 1J,L), consistent with the fSTC data (Fig. 1C–E). This effect is consistent with PAR1 speeding clearance of synaptically-released glutamate from the extracellular space and could be explained by three potential mechanisms: (*1*) TFLLR increases the surface expression of glutamate transporters; (*2*) TFLLR changes the diffusion properties of the entire neuropil; (*3*) TFLLR induces local structural rearrangements of astrocytic processes adjacent to Schaffer collateral synapses. The following sections aim to test each one of these scenarios.

### PAR1 activation does not change the astrocytic glutamate uptake capacity

**Figure 2:**
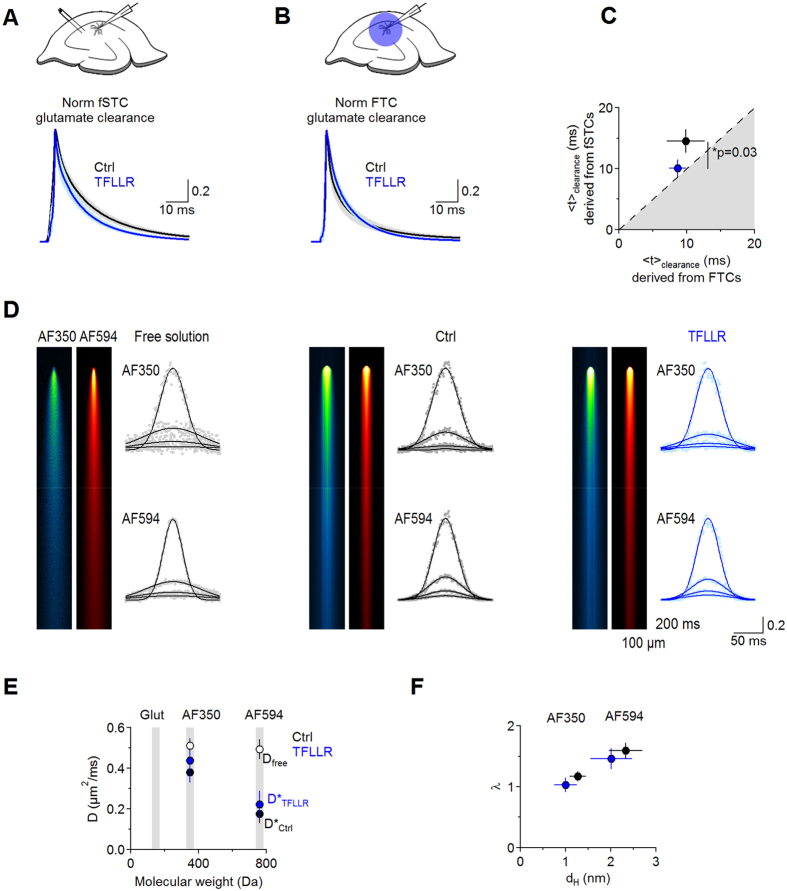
PAR1 activation speeds glutamate clearance without altering the total uptake capacity and diffusion properties of the neuropil. (**A**) *Top:* scheme of the experimental design. Electrical stimuli evoke glutamate release from Schaffer collaterals. Whole-cell patch-clamp recordings from astrocytes in CA1 *stratum radiatum* were used to record transporter currents*. Bottom:* Time course of synaptically-released glutamate clearance in control (*black*) and TFLLR (*blue*). Thick lines and shaded areas represent mean ± S.E.M. (**B**) *Top:* schematic of the experimental design. MNI-L-glutamate (100 μM) was uncaged over the entire field of view to evoke FTCs in astrocytes. *Bottom:* Time course of glutamate clearance derived from FTCs, in control (*black*) and in TFLLR (*blue*). Thick lines and shaded areas represent mean ± S.E.M. fSTCs in (**A**) and FTCs in (**B**) were obtained from the same astrocytes. (**C**) Summary graph: clearance of synaptically-released glutamate is faster in TFLLR (fSTC <t>_clearance_ Ctrl 15.4 ± 1.8 ms (n = 9), TFLLR 10.0 ± 1.3 ms (n = 9) *p = 0.03). Clearance of MNI-L-glutamate is similar in control conditions and in TFLLR, suggesting that PAR1 activation does not alter the astrocytic glutamate uptake capacity (FTC <t>_clearance_ Ctrl 10.7 ± 2.8 ms (n = 9), TFLLR 8.7 ± 1.2 ms (n = 9) *p = *0.50). (**D**) *Left:* kymographs showing the diffusion profile of AF350 and AF594, pressure applied in free solution (*left*), in control slices (*middle*) and in slices treated with TFLLR (*right*). Averages of 10 consecutive applications. *Right:* Gaussian intensity profiles measured 0.7, 2.6, 4.9, and 7.2 s after the pressure application. (**E**) Summary graph: diffusion coefficients of AF350 and AF594 in free solution (*D*_*free AF350*_ 0.51 ± 0.03 μm^2^/ms (n = 6), *D*_*free AF594*_ 0.49 ± 0.05 μm^2^/ms (n = 6)) and in slices in control (D*_AF350_ 0.38 ± 0.05 μm^2^/ms (n = 6), D*_AF594_ 0.17 ± 0.05 μm^2^/ms (n = 6)) and TFLLR (D*_AF350_ 0.44 ± 0.11 μm^2^/ms (n = 6) *p = *0.60, D*_AF594_ 0.22 ± 0.07 μm^2^/ms (n = 6) *p = *0.54). Gray areas highlight the molecular weight of glutamate, AF350 and AF594. (**F**) Summary graph: TFLLR does not alter the hindrance tortuosity (Ctrl *λ*_AF350_ 1.2 ± 0.1 (n = 6), *λ*_AF594_ 1.6 ± 0.1 (n = 5)) (TFLLR *λ*_AF350_ 1.0 ± 0.1 (n = 5) *p = *0.30, *λ*_AF594_ 1.5 ± 0.2 (n = 5) *p = *0.51) and hydrodynamic diameter of AF350 and AF594 in slices (Ctrl *d*_*H* AF350_ 1.3 ± 0.2 (n = 6), *d*_*H* AF594_ 2.3 ± 0.4 (n = 5)) (TFLLR *d*_*H* AF350_ 1.0 ± 0.2 (n = 5) *p = *0.36, *d*_*H* AF594_ 2.0 ± 0.5 (n = 5) *p = *0.56).

If TFLLR increased glutamate transporter expression in astrocytes, we would expect astrocytes to remove flash-uncaged glutamate from the extracellular space faster in TFLLR^20^,^29^. This is because when uncaging glutamate, all transporters are activated simultaneously by the same agonist concentration, regardless of their sub-cellular location and proximity to active synapses. To compare fSTCs and flash-uncaging transporter currents (FTCs) in the same astrocytes, we interleaved synaptic stimulations with UV flashes to uncage a sub-saturating concentration of MNI-L-glutamate in *stratum radiatum* (100 μM; Fig. 2A–C). Consistent with experiments in Fig. 1J,L, clearance of synaptically-released glutamate was faster in TFLLR (<t>_fSTC_ Ctrl 15.4 ± 1.8 ms (n = 9), TFLLR 10.0 ± 1.3 ms (n = 9) *p = 0.03; Fig. 2A,C). In contrast, clearance of flash-uncaged glutamate was similar in control and TFLLR (<t>_FTC_ Ctrl 10.7 ± 2.8 ms (n = 9), TFLLR 8.7 ± 1.2 ms (n = 9) *p* = 0.50; Fig. 2B,C). These findings indicate that PAR1 does not alter the total glutamate uptake capacity of astrocytes and their total glutamate transporter expression.

### PAR1 activation does not change the diffusion properties of the entire hippocampal neuropil

Widespread changes in the diffusion properties of the entire neuropil can be detected by monitoring the decay of the fluorescence signal emitted by pressure-applied, cell-impermeant dyes (Alexa Fluor 350 (AF350), Alexa Fluor 594 (AF594)) with different excitation/emission spectra and molecular weight similar to glutamate (MW_AF350_ = 349 Da, MW_AF594_ = 759, MW_Glut = _147 Da; Fig. 2D–F). We used two-photon laser scanning microscopy (2P-LSM) and line scans to monitor with high temporal resolution the fluorescent intensity profile of AF350 and AF594^32^,^33^, through which we estimated their diffusion coefficient in free solution (*D*_free_) and in stratum radiatum (D*). AF350 and AF594 diffused similarly in control and TFLLR (D*_AF350_ Ctrl 0.38 ± 0.05 μm^2^/ms (n = 6), TFLLR 0.44 ± 0.11 μm^2^/ms (n = 6) *p* = 0.60; D*_AF594_ Ctrl 0.17 ± 0.05 μm^2^/ms (n = 6), TFLLR 0.22 ± 0.07 μm^2^/ms (n = 6) *p* = 0.54; Fig. 2D,E). The hindrance to diffusion experienced by the two fluorophores (i.e. the tortuosity, *λ*) and their effective diameter in aqueous solution (i.e. the hydrodynamic diameter, *d*_*H*_) were also similar in control (*λ*_AF350_ 1.2 ± 0.1 (n = 6), *λ*_AF594_ 1.6 ± 0.1 (n = 5), *d*_*H* AF350_ 1.3 ± 0.2 (n = 6), *d*_*H* AF594_ 2.3 ± 0.4 (n = 5)) and TFLLR (*λ*_AF350_ 1.0 ± 0.1 (n = 5) *p = *0.30; *λ*_AF594_ 1.5 ± 0.2 (n = 5) *p = *0.51; *d*_*H* AF350_ 1.0 ± 0.2 (n = 5) *p = *0.36; *d*_*H* AF594_ 2.0 ± 0.5 (n = 5) *p = *0.56; Fig. 2F). These findings indicate that PAR1 activation does not alter the diffusion properties of small molecules through large regions of the hippocampal neuropil that extend for 50–100 μm, significantly larger than individual Schaffer collateral synapses^34^. Despite its high temporal resolution, the spatial resolution of 2P-LSM in comparison to the size of the extracellular space and may not detect subtle effects on diffusion that occur at the nanometer scale. Therefore, the 2P-LSM diffusion analysis cannot rule out that local and more subtle effects of PAR1 might occur in the immediate vicinity of excitatory synapses.

### PAR1 activation induces local changes in the structure of the neuropil

**Figure 3:**
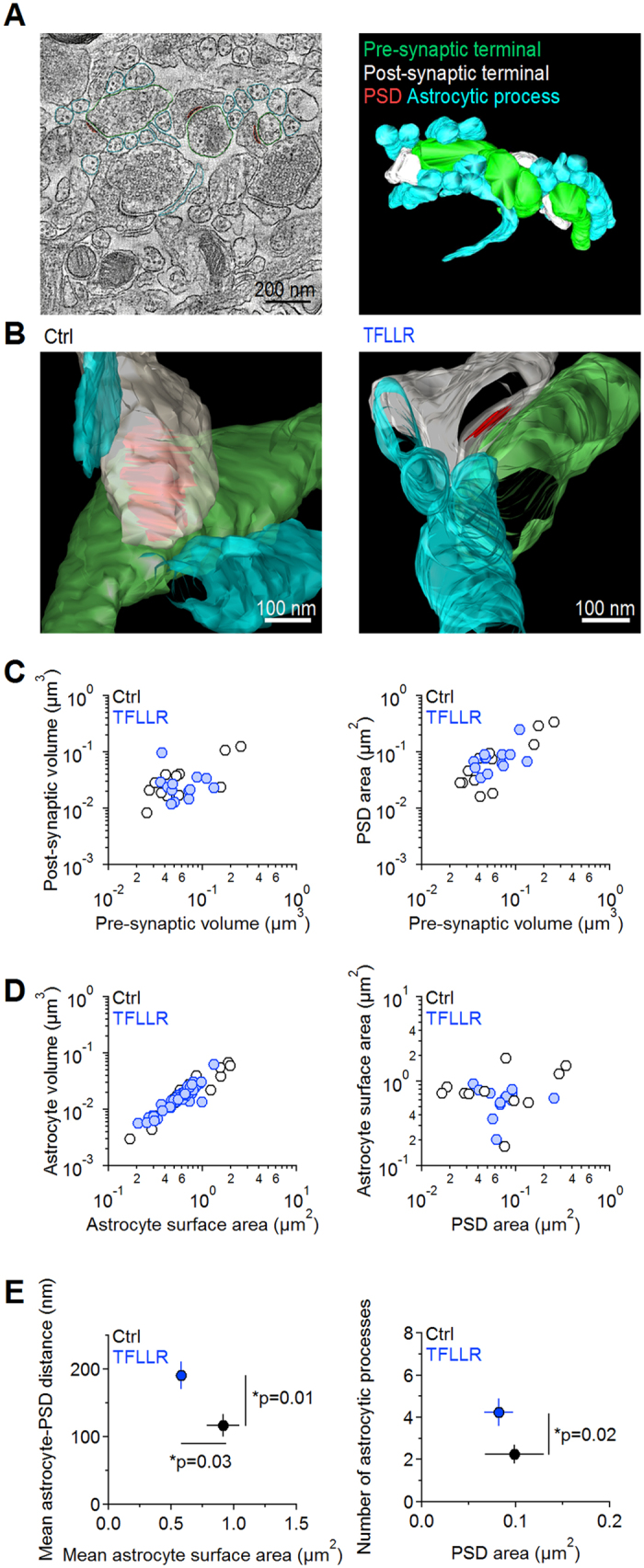
PAR1 activation alters the structure of the neuropil around excitatory synapses. (**A)** *Left:* Electron micrograph (20,000X) showing a 2D image of the hippocampal neuropil in CA1 *stratum radiatum*. Color-coded contour lines were used to manually trace and reconstruct the structure of the pre-synaptic terminal (*green*), post-synaptic terminal (*white*), PSD (red) and astrocytic processes (*light blue*). *Right:* Snapshot of the volumes reconstructed from the contours shown in the left panel. (**B**) Close-up views of a representative synaptic contact reconstructed from control sections (*left*) and sections treated with TFLLR (*right*). (**C**) *Left:* Logarithmic scatter plot of the volume of the post- and pre-synaptic terminals of each synapse reconstructed from control (*white* (n = 12)) and TFLLR-treated slices (*light blue* (n = 13)). *Right:* Logarithmic scatter plot of the PSD area plotted against the volume of the pre-synaptic terminal of each synapse reconstructed from control (*white*) and TFLLR-treated slices (*light blue*). The size of the pre- and post-synaptic terminal increases progressively as the area of the PSD increases, suggesting that each one of these parameters correlates with the size of the entire synaptic contact. (**D**) *Left:* The volume of the peri-synaptic astrocytic processes increases progressively with their surface area. *Right:* lack of correlation between the surface area of astrocytic processes and the PSD area. This finding suggests that under our experimental conditions the astrocytic coverage is similar across synaptic contacts of different dimensions. (**E**) *Left:* the average astrocyte-PSD distance and the mean astrocyte surface area at synapses reconstructed from sections of control slices (*black;* astrocyte-PSD distance 116.5 ± 16.7 nm, mean astrocyte surface area 0.92 ± 0.13 μm^2^ (n = 11)) change in TFLLR (*blue;* astrocyte-PSD distance 190.9 ± 20.7 nm (n = 13) *p = 0.01, mean astrocyte surface area 0.58 ± 0.04 μm^2^ (n = 13) *p = 0.03). *Right:* the average number of astrocytic processes surrounding synapses increases in TFLLR (number of astrocytic processes Ctrl 2.2 ± 0.4, PSD area 0.10 ± 0.03 μm^2^ (n = 12)) (number of astrocytic processes TFLLR 4.2 ± 0.7 *p = 0.02, PSD area 0.08 ± 0.02 μm^2^ (n = 13) *p* = 0.64).

We used axial STEM tomography to obtain higher resolution information on the structure of the neuropil on thick sections (∼1 μm) of *stratum radiatum* from acute hippocampal slices prepared using the same procedures used for the electrophysiology experiments. We analyzed samples from control slices and from slices treated with TFLLR (30 μM) for 30 min (as in the electrophysiology experiments). We manually traced the pre-synaptic terminal, post-synaptic density (PSD), spine head and astrocytic processes around excitatory synapses in the axial STEM tomography data (Fig. 3A,B; Table 1). In agreement with previous findings, the volume of the reconstructed excitatory synapses varied widely across synapses^34^ (Fig. 3C, *Left*). The volume of pre- and post-synaptic terminals was highly correlated with each other, in control and TFLLR (*r* = 0.86; Fig. 3C*, Left*). The PSD area was also highly correlated with the volume of pre-synaptic terminals (*r* = 0.94; Fig. 3C*, Right*), suggesting that any of these parameters can serve as proxy measures for the size of the entire synapse. We confirmed that the volume and surface area of the astrocytes were proportional and correlated to one another (*r* = 0.93; Fig. 3D*, Left*). The astrocyte surface area to volume ratio differed across synapses of different sizes (Fig. 3D***, Left***). Because preserving the surface area to volume ratio as size increases requires changing to more complex shapes, these findings suggest that the overall shape of astrocytic processes does not change depending on their size. We did not detect any correlation between the surface area of astrocytic processes and the surface area of the PSD (Fig. 3D*, Right*) or between the size of a synapse and the distance of neighboring astrocytic processes (data not shown). Because at Schaffer collateral synapses the size of the PSD and of the active zone scales with release probability^34^,^35^, this finding suggests that the extent of astrocytic coverage, in our experimental conditions, is not proportional to synapse release probability. All these general structural features remained unaltered in TFLLR (Fig. 3A–D). Surprisingly, TFLLR caused an increase in the nearest distance between the PSD and neighboring astrocytic processes (Ctrl 116.5 ± 16.7 nm (n = 11), TFLLR 190.9 ± 20.7 nm (n = 13) *p = 0.010; Fig. 3E*, Left*) and a reduction in the astrocyte mean surface area (Ctrl 0.92 ± 0.13 μm^2^ (n = 11), TFLLR 0.58 ± 0.04 μm^2^ (n = 13) *p = 0.029; Fig. 3E*, Left*). These findings are not in conflict with the lack of effect of TFLLR on cell capacitance which, at its best, provides information on the capacitance and surface area of entire cells, not of their finest processes (Fig. 1B). They are surprising however, because by allowing astrocytic processes to move away from excitatory synapses and shrink, PAR1 should *prolong* – not *speed* – glutamate clearance (Fig. 1J,L). One important additional effect of TFLLR is that it causes proliferation of astrocytic processes, increasing their number around excitatory synapses (Ctrl 2.2 ± 0.4 (n = 12), TFLLR 4.2 ± 0.7 (n = 13) *p = 0.022) without altering the PSD area (Ctrl 0.099 ± 0.03 μm^2^ (n = 12), TFLLR 0.08 ± 0.02 μm^2^ (n = 13) *p* = 0.64; Fig. 3E*, Right*). On its own, this effect would increase the local glutamate uptake capacity of astrocytes and speed glutamate clearance. Together, these data shows that PAR1 induces rapid and complex changes in the local structure of the neuropil around excitatory synapses. It is difficult, however, to predict whether all these effects should speed or slow down glutamate clearance.

### In silico models recapitulate the effect of PAR1 activation on glutamate clearance

**Figure 4:**
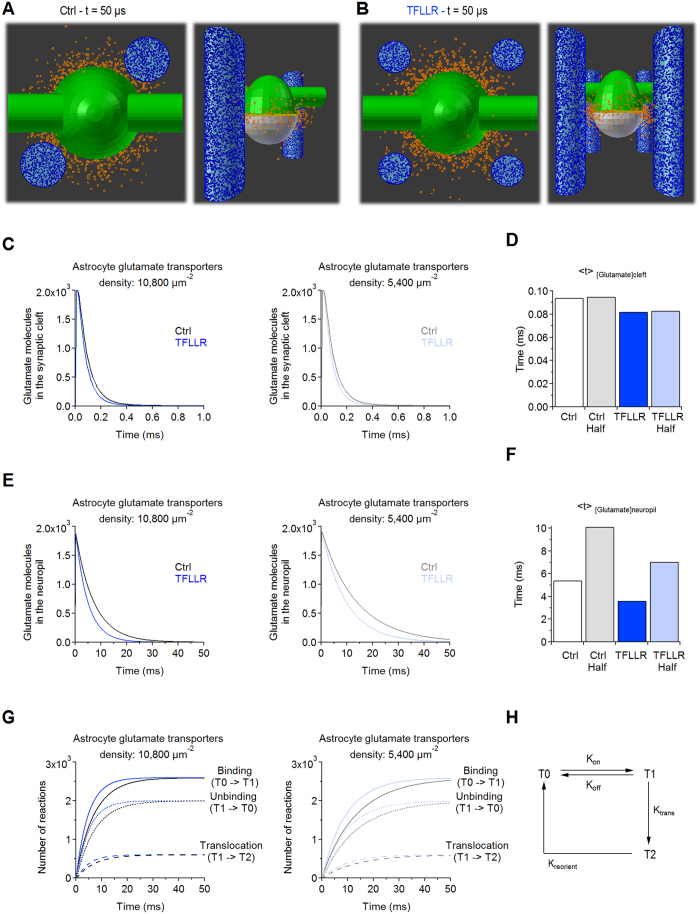
Local changes in astrocytic coverage of excitatory synapses speed the lifetime of synaptically-released glutamate in the extracellular space (simplified geometries). (**A**,**B**) Snapshots of the geometries used to run 3D Monte Carlo reaction-diffusion simulations in control (**A**) and TFLLR (**B**), taken 50 μs after the release of 2,000 glutamate molecules from the center of the synaptic cleft. The size of the pre- (*green*) and post-synaptic terminal (*white*), the number and surface area of neighboring astrocytic processes (*light blue*) and their distance from the PSD match the average values from the axial STEM tomography reconstructions (Fig. 3, Table 1 and 2). Glutamate transporters are represented as icosahedrons (*blue;* 10,800/μm^2^)^13^; glutamate molecules are represented as tori, diffusing inside (*yellow*) and outside the synaptic cleft (*orange*). (**C**) *Left:* Time course of glutamate diffusion in the synaptic cleft in control (*black*) and TFLLR (*blue*). *Right:* As in the left panel, with reduced surface density of glutamate transporters (5,400/μm^2^). (**D**) Summary graph: TFLLR speeds the glutamate concentration profile in the cleft, at different glutamate transporter densities. (**E**) Time course of glutamate diffusion in the extra-synaptic neuropil, with an astrocytic glutamate transporter surface density of 10,800 μm^−2^ (*left*) and 5,400/μm^2^ (*right*). (**F**) Summary graph: The lifetime of glutamate in the neuropil is faster in TFLLR than in control conditions, at different glutamate transporter densities. (**G**) Cumulative plots of binding (*solid curves*), unbinding (*dotted curves*) and translocation reactions (*dashed curves*) measured in control (*black*) and TFLLR (*blue*), with 10,800/μm^2^ (*left*) and 5,400/μm^2^ glutamate transporters (*right*). (**H**) Simplified Markov model of glutamate transporters used in the 3D Monte Carlo reaction-diffusion simulations (rate constants for each reaction are reported in the Methods).

**Figure 5:**
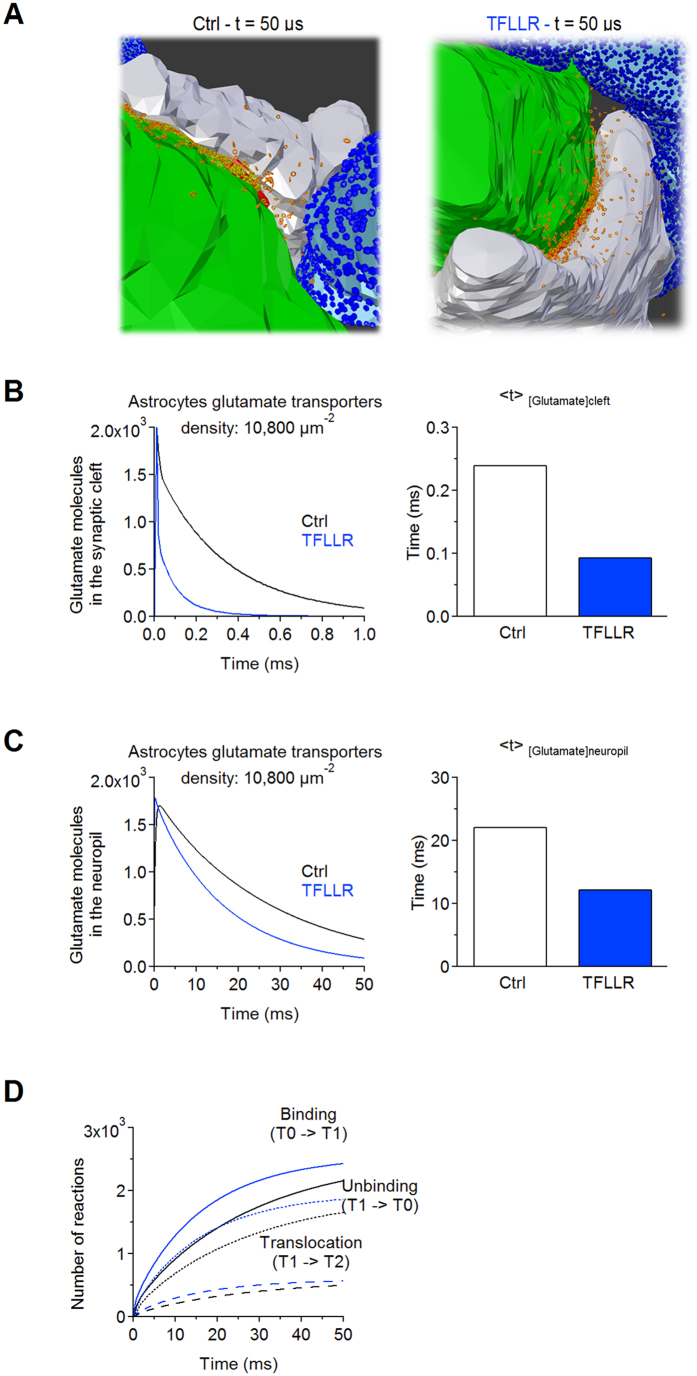
Local changes in astrocytic coverage of excitatory synapses speed the lifetime of synaptically-released glutamate in the extracellular space (reconstructed geometries). (**A**) Close-up view of glutamate molecules (*orange*) diffusing away from the synaptic cleft in a 3D reconstruction from axial STEM tomography of an excitatory synapse in control (*left*) and TFLLR (*right*). The snapshots are taken 50 μs after glutamate release from the center of the synaptic cleft. Color codes: pre-synaptic terminal (*green*), post-synaptic terminal (*white*), PSD (*red*), astrocytic processes (*light blue*), glutamate transporters (*blue icosahedrons*), extra-synaptic glutamate (*orange tori*), cleft glutamate (*yellow tori*). The geometrical parameters of these synapses are included in Table 3. (**B**) L*eft:* Time course of glutamate diffusion in the synaptic cleft, in control (*black*) and TFLLR (*blue*). *Right:* Summary graph showing that TFLLR speeds the lifetime of glutamate in the synaptic cleft. (**C**) *Left:* Time course of glutamate diffusion in the neuropil in control (*black*) and TFLLR (*blue*). *Right:* Summary graph showing that TFLLR speeds the lifetime of glutamate in the neuropil. (**D**) Cumulative plots of binding (*solid curves*), unbinding (*dotted curves*) and translocation reactions (*dashed curves*) measured in control (*black*) and TFLLR (*blue*) simulations.

As mentioned in the previous section, *local* changes in the number and surface area of astrocytic processes could increase the *local* uptake capacity of the neuropil at excitatory synapses. A rough estimate of the local uptake capacity can be obtained by multiplying the mean surface density of glutamate transporters (*d* = 10,800 μm^−2^)^13^ by the surface area of one astrocytic process (SA_1astro_) and the number of astrocytic processes at each synapse (N_astro_). In our experiments, this leads to a ∼40% increase in the local uptake capacity of the neuropil in TFLLR. To determine how these effects could act in concert with changes in the distance between astrocytes and synapses, we generated 3D Monte Carlo reaction-diffusion simulations of glutamate diffusion in the hippocampal neuropil. We first used simplified geometries, where the volume and surface area of neuronal and astrocytic structures matched those obtained from the reconstructions (Fig. 4A,B, Table 2). We confirmed that these simulations are sensitive to changes in glutamate uptake capacity, because the lifetime of glutamate in the neuropil is prolonged when reducing the surface density of glutamate transporters (Fig. 4C–H). In these simulations, the extracellular glutamate concentration decays faster in TFLLR than in control, in the cleft (Fig. 4C* Left,D*) and the neuropil (Fig. 4E* Left,F*), even when the uptake capacity is low (Fig. 4C* Right,D,E Right,F*). This effect is associated with an increased rate of glutamate binding, unbinding and translocation by astrocytic transporters in TFLLR (Fig. 4G,H). These results are consistent with our experimental findings (Fig. 1H,I). To rule out any bias due to the use of simplified geometries, we performed analogous simulations using geometries from the axial STEM tomography reconstructions (Fig. 5; Table 3). Here the PSD-astrocytes distance, number of astrocytic processes and astrocyte surface area closely matched the mean values measured across synapses in control and TFLLR (Fig. 3E). The simulations provided similar results to those in Fig. 4, showing that TFLLR speeds the lifetime of glutamate in the cleft and the neuropil (Fig. 5). The effects of TFLLR were more pronounced when using reconstructed synapses, likely because the synaptic cleft is less confined than in simplified geometries. The results of these simulations support the hypothesis that PAR1, by modifying the 3D organization of astrocytic processes at excitatory synapses, speeds clearance of synaptically-released glutamate.

### PAR1 activation reduces GluA receptor activation in CA1 pyramidal cells

**Figure 6:**
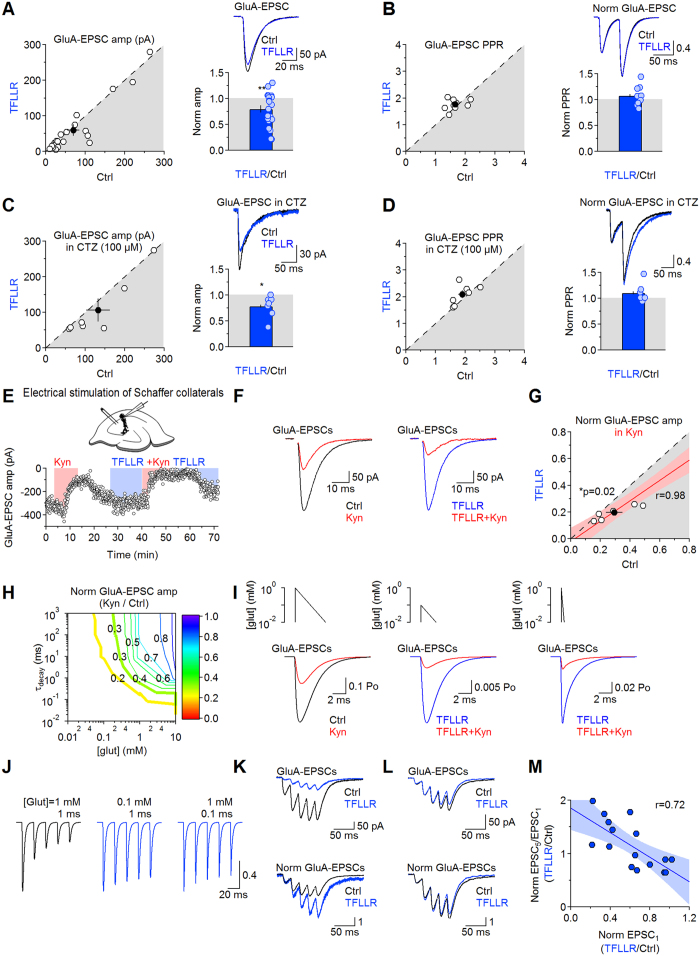
PAR1 activation reduces GluA activation. (**A)** *Left:* GluA-EPSC amplitude in control (70.4 ± 15.4 pA) and TFLLR (59.1 ± 15.8 pA (n = 21) *p* = 0.06). *Right top:* example GluA-EPSCs. *Right bottom:* TFLLR reduces the GluA-EPSC amplitude (0.79 ± 0.07 (n = 21) **p = 0.008). (**B**) *Left:* GluA-EPSC PPR in control (1.66 ± 0.10) and TFLLR (1.76 ± 0.07 (n = 9) *p* = 0.39). *Right top:* example paired GluA-EPSCs. *Right bottom:* TFLLR does not change PPR (1.08 ± 0.06 (n = 9) *p* = 0.26). (**C**) As in A, in cyclothiazide (CTZ 100 μM; Ctrl132.4 ± 30.11 pA; TFLLR 106.5 ± 32.0 pA (n = 7) *p* = 0.07). TFLLR reduces the GluA-EPSC amplitude in CTZ (0.78 ± 0.08 (n = 7) *p = 0.03). **(D)** AS in B, in CTZ. GluA-EPSC PPR in control (1.90 ± 0.13) and TFLLR (2.08 ± 0.14 (n = 7) *p* = 0.19). TFLLR does not change PPR in CTZ (1.10 ± 0.02 (n = 7) *p* = 0.17). (**E**) *Top:* scheme of the experimental design. *Bottom:* time course of GluA-EPSC amplitude. GluA-EPSCs are recorded in control, then in kynurenate (300 μM). Kynurenate was washed out until the GluA-EPSC amplitude recovered to its baseline value. Then we applied TFLLR, washed in kynurenate in TFLLR and returned to TFLLR. **(F**) *Left:* GluA-EPSCs in control (*black*) and kynurenate (*red*). *Right:* same cell, GluA-EPSCs in TFLLR (*blue*) and in TFLLR + kynurenate (*red*). **(G)** Summary graph: GluA-EPSCs are blocked more effectively by kynurenate in TFLLR (Ctrl 0.29 ± 0.06; TFLLR 0.19 ± 0.02 (n = 7) *p = 0.02). Linear regression (*red*), 95% confidence bands (*pink*) and Pearson’s correlation coefficient (*r*) are shown in the graph. (**H**) Contour plot of the normalized GluA-EPSC amplitude in kynurenate (300 μM). Simulated GluA-EPSC were evoked by glutamate transients with different peak concentrations and decay. Thick lines match the kynurenate block observed experimentally, in control (*green*) and TFLLR (*yellow*). (**I**) Simulated GluA-EPSC kynurenate-block. (**J**) Simulated GluA-EPSC trains evoked by different glutamate transients. (**K**) T*op:* GluA-EPSCs in control (*black*) and TFLLR (*blue*). TFLLR reduced the amplitude of the first GluA-EPSC to 12%. *Bottom:* GluA-EPSCs normalized by the amplitude of the first GluA-EPSC. (**L**) As in K, for a cell in which TFLLR reduced the amplitude of the first GluA-EPSC to 86%. (**M**) Summary graph: the larger the GluA-EPSC block by TFLLR, the larger is summation of consecutive GluA-EPSCs. Linear regression (*blue*), 95% confidence bands (*light blue*) and *r* are shown in the graph.

If PAR1 activation speeds glutamate clearance from astrocytes, we would expect it to also change glutamatergic signaling among neurons mediated by GluA/N receptors. Accordingly, TFLLR (30 μM) induced a consistent decrease in the GluA-EPSC amplitude in CA1 pyramidal cells voltage-clamped at −70 mV (0.79 ± 0.07 (n = 21) **p = 8.0e-3; Fig. 6A). This effect was not associated with changes in the paired-pulse ratio (PPR, 1.08 ± 0.06 (n = 9) *p* = 0.26; Fig. 6B), ruling out pre-synaptic effects of PAR1^36^,^37^. To rule out any confounding effect due to GluA receptor desensitization, we repeated these experiments in cyclothiazide (100 μM)^38^,^39^,^40^,^41^ (Fig. 6C,D). In cyclothiazide, TFLLR reduced the GluA-EPSC amplitude (0.78 ± 0.08 (n = 7) *p = 0.03), not PPR (1.10 ± 0.02 (n = 7) *p* = 0.17). Therefore, PAR1 reduces GluA receptor activation without altering pre-synaptic function.

If PAR1 sped glutamate clearance, we would also expect it to increase the sensitivity of GluA receptors to the competitive, low-affinity antagonist kynurenate, because its ability to rapidly bind and unbind from GluA receptors varies with the amplitude and decay of the glutamate concentration profile detected by GluA receptors^19^,^29^,^42^,^43^,^44^. To test this hypothesis, we recorded GluA-EPSCs in control and kynurenate (300 μM). We then washed out kynurenate, applied TFLLR, repeated the kynurenate application in TFLLR and then washed out kynurenate (Fig. 6E). Kynurenate reduced the GluA-EPSC amplitude to 0.29 ± 0.06 in control (***p = 3.8e-3; Fig. 6E–G) and 0.19 ± 0.02 in TFLLR ((n = 7) ***p = 4.1e-4; Ctrl vs TFLLR *p = 0.02). The effects of kynurenate in control and TFLLR were highly correlated (*r* = 0.98; Fig. 6G), suggesting that synapses activated by smaller glutamate transients (not necessarily smaller synapses) are also more susceptible to changes in astrocytic coverage induced by TFLLR. Kinetic models of GluA receptors confirmed that the more pronounced effect of kynurenate on GluA-EPSCs in TFLLR is consistent with PAR1 activation reducing the amplitude and/or duration of the glutamate concentration profile at GluA receptors (Fig. 6H,I).

The ability of TFLLR to reduce GluA receptor activation could shape the response of excitatory synapses to repetitive stimuli, as indicated by kinetic models in which reducing the peak/decay of the glutamate concentration profile increases summation of consecutive GluA-EPSCs (Fig. 6J). Cells in which GluA-EPSCs were more powerfully blocked by TFLLR showed increased summation of consecutive EPSCs (Fig. 6K,M). Together, these results suggest that the subtle structural changes in the local environment around excitatory synapses induced by PAR1 can significantly change the dynamics of excitatory synaptic transmission at Schaffer collateral synapses.

### PAR1 activation reduces phasic GluN receptor activation in CA1 pyramidal cells

**Figure 7:**
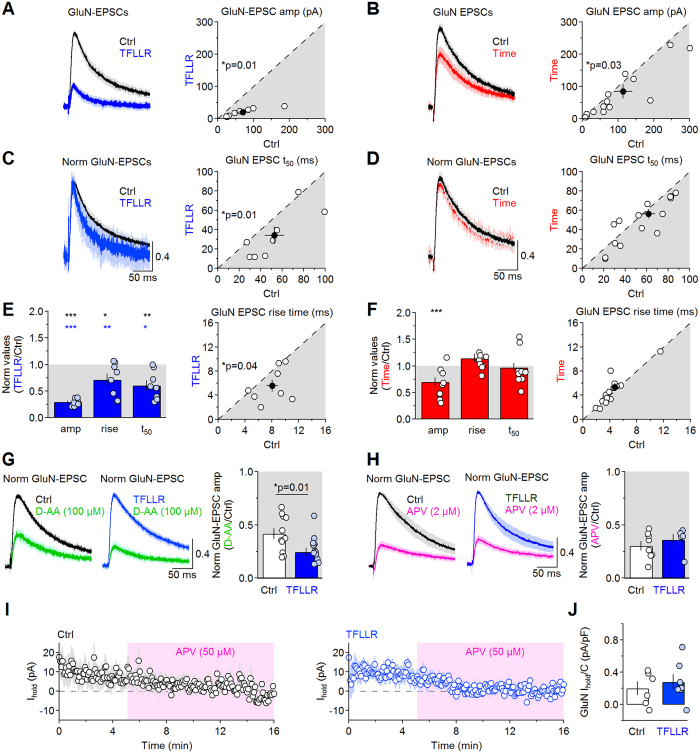
PAR1 activation reduces GluN activation. (**A**) *Left:* GluN-EPSCs in control (*black*) and TFLLR (*blue*), normalized by the GluN-EPSC peak in control. *Right:* GluN-EPSC amplitude in control (69.8 ± 19.2 pA) and TFLLR (19.8 ± 4.4 pA (n = 8) *p = 0.01). (**B**) *Left:* As in A, for GluN-EPSCs in control (*black*) and after 30 min recording time (*red*). *Right:* GluN-EPSC amp in control (114.4 ± 25.8 pA) and after 30 min (84.7 ± 22.0 pA (n = 12) *p = 0.03). (**C**) *Left:* Peak-normalized GluN-EPSCs in control (*black*) and TFLLR (*blue*). *Right:* GluN-EPSC t_50_ in control (52.6 ± 9.0 ms) and TFLLR (34.2 ± 9.0 ms (n = 8) *p = 0.01). (**D**) As in C, for time dependent control (Ctrl: 61.7 ± 6.2 ms, Time: 56.3 ± 4.9 ms (n = 12) *p* = 0.20). (**E**) *Left:* TFLLR reduces the GluN-EPSC amplitude, rise and decay time (Norm GluN-EPSC amp 0.29 ± 0.02 ***p = 1.1e-8, TFLLR vs Time ***p = 2.4e-4; Norm GluN-EPSC 20–80% rise 0.71 ± 0.11 *p = 0.04, TFLLR vs Time **p = 7.3e-3; Norm GluN-EPSC t_50_ 0.61 ± 0.09 (n = 8) **p = 0.004, TFLLR vs Time *p = 0.01). *Right:* GluN-EPSC 20–80% rise in control (8.1 ± 0.8 ms) and TFLLR (5.6 ± 1.0 ms (n = 8) *p = 0.04). (**F**) As in E, for time-dependent control. *Left:* (Norm GluN-EPSC amp 0.70 ± 0.08 ***p = 0.003; Norm GluN-EPSC 20–80% rise 1.14 ± 0.08, *p* = 0.10; Norm GluN-EPSC t_50_ 0.971 ± 0.08 (n = 12), *p* = 0.69). *Right:* (GluN-EPSC 20–80% rise 4.7 ± 0.6 ms, TFLLR 5.3 ± 0.7 ms (n = 12) *p* = 0.11. (**G**) *Left:* GluN-EPSCs in control (*black*) or TFLLR (*blue*) and after D-AA (100 μM; *green*). *Right:* D-AA reduces the GluN-EPSC amplitude more in TFLLR (Norm GluN-EPSC amp Ctrl 0.42 ± 0.05 ***p = 4.5e-7; TFLLR 0.25 ± 0.04 ***p = 2.8e-11; Ctrl vs TFLLR *p = 0.01). **(H)** *Left:* GluN-EPSCs in control (*black*) or TFLLR (*blue*) and after APV (2 μM; *magenta*). *Right:* As in K, for APV (Norm GluN-EPSC amp Ctrl 0.30 ± 0.04 ***p = 9.4e-7; TFLLR: 0.36 ± 0.05 ***p = 2.7e-4; Ctrl vs TFLLR *p* = 0.43). (**I**) Time course of the holding current (I_hold_) in control (*left*) or in TFLLR (*right*) at a holding potential of 40 mV before and after blocking GluN receptors with APV (50 μM). The pink shaded area represents the duration of the APV application. (**J**) Summary graph: The GluN holding current density, measured as the ratio of the APV-sensitive holding current and the cell capacitance, is similar in control (69.8 ± 19.2 pA) and TFLLR (0.28 ± 0.09 pA/pF (n = 8) *p* = 0.53).

The slow glutamate unbinding and desensitization rates of GluN receptors make them well-suited to detect PAR1-induced changes in phasic and tonic glutamatergic transmission^45^,^46^. TFLLR reduced the amplitude, rise and decay of GluN-EPSCs (amp 0.29 ± 0.02 ***p = 1.1e-8, 20–80% rise 0.71 ± 0.11 *p = 0.04, t_50_ 0.61 ± 0.09 (n = 8) **p = 4.0e-3; Fig. 7A,C,E). These effects were not accounted by rundown of GluN-EPSCs, which we monitored by recording GluN-EPSCs for ∼30 min without TFLLR (Fig. 7B,D,F). In these time-dependent control experiments, there was a small reduction of the GluN-EPSC amplitude, with no change in its rise and decay (amp 0.70 ± 0.08, ***p = 0.003; 20–80% rise 1.14 ± 0.08, *p* = 0.10; t_50_ 0.971 ± 0.08 (n = 12) *p* = 0.69). However, the effects of TFLLR on GluN-EPSCs were significantly different from the ones measured in time-dependent control experiments (amp ***p = 2.4e-4; 20–80% rise **p = 7.3e-3; t_50_ *p = 0.01).

If these effects were due to faster glutamate clearance (Fig. 1J), we would expect D-AA, a competitive low-affinity GluN receptor antagonist, to reduce the GluN-EPSC amplitude more in TFLLR than in control. Accordingly, D-AA (100 μM) reduced the GluN-EPSC amplitude to 0.42 ± 0.05 in control ((n = 11) ***p = 4.5e-7) and 0.25 ± 0.04 in TFLLR ((n = 14) ***p = 2.8e-11; Ctrl vs TFLLR *p = 0.014). In contrast, the competitive high-affinity antagonist APV reduced the GluN-EPSC amplitude similarly in control (0.30 ± 0.04 ***p = 9.4e-7) and TFLLR (0.36 ± 0.05 ***p = 2.7e-4; Ctrl vs TFLLR *p* = 0.43; Fig. 7G,H). The different effect of D-AA in control and TFLLR, not detected with APV, suggests that PAR1 activation reduces GluN-receptor activation by altering the glutamate concentration profile at GluN receptors.

Does TFLLR also change the steady-state concentration of glutamate in the extracellular space? A tonic GluN-mediated current can be measured by voltage-clamping CA1 pyramidal cells at + 40 mV, before and after blocking GluN receptors with a saturating concentration of APV (50 μM)^47^. For comparison across cells with different surface area, we divided the APV-sensitive tonic current by the cell capacitance^48^. The GluN tonic current density was similar in control (0.20 ± 0.08 pA/pF (n = 6)) and TFLLR (0.28 ± 0.09 pA/pF (n = 8) *p* = 0.53; Fig. 7J). These findings are consistent with the hypothesis that PAR1 activation controls phasic synaptic transmission without altering the steady-state ambient glutamate concentration.

### PAR1 activation impairs long-term potentiation

**Figure 8:**
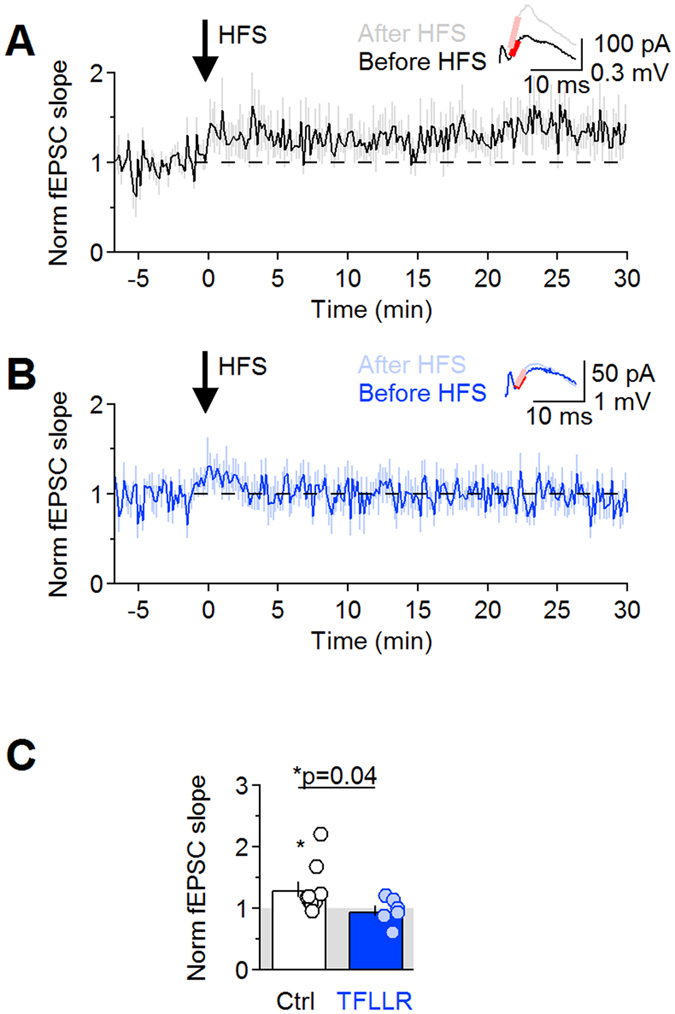
PAR1 activation impairs LTP. (**A**) Time course of baseline-normalized fEPSC slope before and after LTP induction with a high-frequency stimulation (HFS) protocol (100 Hz, 1 s), in control. (**B**) As in A, in TFLLR. (**C**) Summary of changes in the EPSC slope induced the by HFS in control (Norm fEPSC slope Ctrl: 1.31 ± 0.13 (n = 9) *p = 0.04) and TFLLR (0.96 ± 0.09 (n = 6) *p* = 0.66; Ctrl vs TFLLR *p = 0.04).

GluA/N receptors play a crucial role in shaping long-term plasticity at Shaffer collateral synapses^49^. LTP induction requires a rise in post-synaptic calcium concentration through GluN receptors, whereas LTP expression requires trafficking of GluA receptors to the cell membrane^49^. We performed extracellular field recordings from CA1 *stratum radiatum* and used a high-frequency stimulation (HFS: 100 Hz, 1 s) as the LTP-induction protocol. HSF induced LTP in control conditions (Norm fEPSC slope 1.31 ± 0.13 (n = 9) *p = 0.04; Fig. 8A,C) but did not induce LTP in TFLLR (Norm fEPSC slope 0.96 ± 0.09 (n = 6) *p* = 0.66; Ctrl vs TFLLR *p = 0.04 Fig. 8B,C). Therefore, by inducing local changes in the local structure of the neuropil surrounding excitatory synapses, PAR1 speeds glutamate clearance, limits GluA/N receptor activation and impairs LTP expression at Schaffer collateral synapses.

## Discussion

Astrocytes play a fundamental role in clearing synaptically-released glutamate from the extracellular space, through the activity of glutamate transporters^18^,^42^,^50^. By rapidly binding synaptically-released glutamate, the transporters limit glutamate diffusion away from the synaptic cleft and keep the extracellular glutamate concentration at low nanomolar levels, to prevent glutamate-induced excitotoxicity^20^,^29^,^47^,^51^. Recent findings indicate that the fine astrocytic processes adjacent to excitatory synapses are highly dynamic and undergo significant structural reorganization in physiological conditions and during hypotonic stress^21^,^22^,^52^,^53^,^54^. To date, no experimental evidence has shown that these structural rearrangements can be regulated by activation of membrane serine protease, G protein-coupled receptors like PAR1. The results presented here describe a novel form of regulation of astrocytic processes around excitatory synapses and of glutamate uptake, both mediated by PAR1 activation. We show that the rapid and complex structural reorganization of astrocytic processes induced by PAR1 shape the lifetime of synaptically-release glutamate in the extracellular space (Figs 1 and 2), phasic excitatory synaptic transmission (Figs 6 and 7) and long-term plasticity (Fig. 8). They do not alter the overall uptake capacity of the entire hippocampal neuropil (Fig. 2), and do not lead to changes in tonic glutamatergic signaling (Fig. 7).

PAR1 is a G-protein coupled receptor known for its role in hemostasis and inflammation^6^,^55^. In the central nervous system, PAR1 is mostly expressed in astrocytic cell bodies and fine processes closely opposed to excitatory synapses and blood brain capillaries^1^. PAR1 interacts with G proteins of the Gq/11, Gi/o and G12/13 subfamilies, which bind to distinct cytoplasmic domains of the receptor^7^. Our findings indicate that PAR1 activation does not change the total uptake capacity of astrocytes and the diffusion properties of the entire hippocampal neuropil (Fig. 2). Instead, PAR1 activation has profound *local* effects and changes the fine structure and arrangement of astrocytic processes around excitatory synapses, which include reduced surface area, increased distance from the synapse and proliferation of astrocytic processes (Fig. 3). These findings are consistent with previous work suggesting the existence of a PAR1-dependent control of astrocytic processes proliferation, based on GFAP immuno-labeling experiments^56^. This work, however, did not clarify whether this effect was associated with functional changes in glutamate clearance^56^. Our findings are consistent with a growing number of reports suggesting that the precise location of astrocytic processes with respect to excitatory synapses powerfully controls the rate of glutamate uptake^50^,^53^,^54^,^57^,^58^. They show that these changes can occur rapidly, within 30 min following PAR1 activation. The reason for such a local effect of PAR1 is not known, but may depend on the sub-cellular distribution of target molecules. Accordingly, the fact that astrocytic processes express high levels of target glutamate transporters^59^ may render them more susceptible to PAR1 activation.

Our findings do not support other studies suggesting that PAR1 activation triggers glutamate release from astrocytes in the nucleus of the solitary tract, hippocampus and in culture through mechanisms that might involve activation of astrocytic Bestrophin-1 anion channels^60^,^61^,^62^. Not only, we find that PAR1 speeds glutamate clearance, but it also reduces GluA/N activation in CA1 pyramidal neurons (Figs 6 and 7). In addition, kynurenate and D-AA (competitive, low-affinity antagonists for GluA and GluN receptors, respectively), which competes with glutamate for binding to their target receptors, reduces the GluA/N EPSC amplitude more effectively in TFLLR than in control conditions, a result that is not consistent with PAR1 activation triggering glutamate release from astrocytes. Our results indicate that the effects of PAR1 influence the glutamate lifetime in and out of the synaptic cleft. The reasons for the discrepancy among studies are not clear, but we cannot rule out the existence of brain region-specific regulatory mechanisms underlying different effects of PAR1 activation. Other technical aspects, including differences in the age of the animals used for the experiments (8–9 weeks in^61^ versus 2–3 weeks in our experiments), might contribute to a progressively lager role of Bestrophin-1 receptors during postnatal development^63^.

The PAR1-dependent modulation of astrocyte morphology and glutamatergic signaling is significant for understanding the function of the brain in pathological conditions. PAR1 is activated by serine proteases in the bloodstream (e.g. thrombin and plasmin), which take part in signaling cascades involved in the formation of blood clots. Under physiological conditions, the blood brain barrier prevents these proteins from diffusing out of the lumen of blood vessels. However, during cerebrovascular damage induced by traumatic brain injury, seizures and hemorrhagic stroke, the blood-brain barrier breaks down leading to diffusion of thrombin and plasmin in the brain neuropil. By reducing the lifetime of glutamate in the extracellular space and by limiting glutamate receptor activation, PAR1 activation induces changes in short-term plasticity at Schaffer collateral synapses, altering their response to repetitive stimuli. PAR1 activation also impairs the expression of LTP, a candidate mechanism underlying memory formation^64^,^65^. In this context, our findings identify PAR1 as an important novel target molecule to prevent the onset of cognitive impairment following small hemorrhagic stroke in the brain.

## Materials and Methods

### Ethics statement

All experimental protocols involving animals were performed in accordance with the guidelines of the Institutional Animal Care and Use Committee at SUNY Albany. The experimental protocols and methods were approved by the Institutional Animal Care and Use Committee at SUNY Albany (Project #13-011).

### Electrophysiology experiments and data analysis

Acute hippocampal slices were obtained from C57BL/6 mice of either sex (P11-18), deeply anesthetized with isoflurane and decapitated in accordance with SUNY Albany Animal Care and Use Committee guidelines. The brain was rapidly removed and placed in ice-cold slicing solution bubbled with 95% O_2_-5% CO_2,_ containing (in mM): 119 NaCl, 2.5 KCl, 0.5 CaCl_2_, 1.3 MgSO_4_·H_2_O, 4 MgCl_2_, 26.2 NaHCO_3_, 1 NaH_2_PO_4_, and 22 glucose; 320 mOsm; pH 7.4. Transverse hippocampal slices (250 μm thick) were prepared using a vibrating blade microtome (VT1200S, Leica Microsystems, Wetzlar, Germany). After cutting, slices were stored in this solution in a submersion chamber at 36 °C for 30 min and at room temperature for at least 30 min and up to 4 hours. The recording solution contained (in mM): 119 NaCl, 2.5 KCl, 2.5 CaCl_2_, 1 MgCl_2_, 26.2 NaHCO_3_, 1 NaH_2_PO_4_, 22 glucose; 300 mOsm; pH 7.4. Whole-cell patch recordings were obtained from CA1 pyramidal neurons and astrocytes identified under infrared-differential interference contrast using an upright fixed-stage microscope (BX51 WI, Olympus Corporation, Tokyo, Japan). The internal solution used to record GluA-EPSCs in CA1 pyramidal cells contained (in mM): 120 CsCH_3_SO_3_, 10 EGTA, 20 HEPES, 2 MgATP, 0.2 NaGTP, 5 QX-314Br, 5 NaCl; 290 mOsm; pH 7.2. The internal solution used to record transporter currents in astrocytes had the same composition except that 120 CsCH_3_SO_3_ was replaced with 120 KCH_3_SO_3_. To record transporter currents, the following drugs were added to the recording solution (in μM): picrotoxin (100), CGP52432 (5), 2,3-Dioxo-6-nitro-1,2,3,4-tetrahydrobenzo[f]quinoxaline-7-sulfonamide (NBQX, 10), (RS)-3-(2-Carboxypiperazin-4-yl)-propyl-1-phosphonic acid (CPP, 10), (2S)-2-Amino-2-[(1S,2S)-2-carboxycycloprop-1-yl]-3-(xanth-9-yl) propanoic acid (LY341495, 1), (RS)-α-Methylserine-O-phosphate (MSOP, 100) and 8-Cyclopentyl-1,3-dipropylxanthine (DPCPX, 1), to block GABA_A_, GABA_B_, GluA, GluN, mGluRII-III and adenosine receptors, respectively. CPP or NBQX were omitted from the recording solution when recording GluA or GluN-EPSCs, respectively. CPP and NBQX were not used to record field EPSPs (Fig. 8). All recordings were obtained under voltage-clamp configuration using a Multiclamp 700B amplifier and a 10 KHz low-pass filter (Molecular Devices, Sunnyvale, CA). All traces were digitized at 10 KHz and analyzed off-line with custom-made software (A.S.) written in IgorPro 6.36 (Wavemetrics, Lake Oswego, OR). The electrode resistance was ∼2.5 MΩ. The series resistance was monitored throughout the experiments by applying a −3 mV pulse, 100 ms before evoking the synaptic currents. Data were discarded if the series resistance changed more than 20% during the course of the experiment. GluA-EPSCs and transporter currents were evoked by delivering constant voltage electrical pulses (50 μs) through bipolar stainless steel electrodes (Cat. MX21AES (JD3); Frederick Haer Company, Bowdoin, ME) placed in CA1 *stratum radiatum*, ∼100 μm away from the recorded cell. Single and paired pulses (100 ms inter-pulse interval) were delivered every 10 s. The amplitude of the sustained K^+^ current was measured in a 50 ms time window, positioned 200 ms after the stimulus artifact. To record flash-activated transporter currents (FTCs), 4-methoxy-7-nitroindolinyl-caged-L-glutamate (MNI-L-glutamate; 100 μM) was added to the perfusion solution and uncaged using a Flashmic Xe-lamp (Rapp OptoElectronic GmbH, Hamburg, Germany) connected to the epi-illumination port^29^. This concentration of MNI-L-glutamate does not saturate glutamate transporters, a necessary requirement to perform the deconvolution analysis and estimate glutamate clearance from FTCs and the results are not expected to differ when uncaging different sub-saturating concentrations of this compound^20^. To subtract the stimulation artifact from the FTC, we recorded responses with the light path blocked. In these experiments, we interleaved single synaptic stimulation, flash, paired synaptic stimulation, flash with light path blocked and delivered each one of them every 10 s. Analysis of transporter currents was performed as described previously^28^,^29^,^66^. All drugs were purchased from Sigma-Aldrich (St. Louis, MO), Tocris Bioscience and Hello Bio (Bristol, UK). TFLLR, with C-terminal amidation, was purchased from Genscript Biotech Corporation (Piscataway Township, NJ). All experiments were performed at room temperature (23–26 °C).

To analyze the facilitated portion of the STCs (fSTCs) and estimate the fast and slow component of the decaying phase (τ_fast_ and τ_slow_, respectively; Supp. Fig. 1, Figs 1 and 2), we fitted the fSTC waveform with the multi-exponential equation:


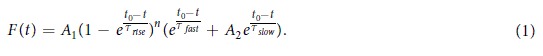


### Two-photon microscopy diffusion analysis

We estimated the diffusion profile of two cell-impermeable fluorophores, Alexa Fluor 350 (AF350 0.2 mM; Cat.A10439, Life Technologies, Carlsbad, CA) and Alexa Fluor 594 (AF594 0.1 mM; Cat.A10438, Life Technologies), with molecular weights (349 and 759 Da, respectively) similar to that of glutamate (147 Da) (Savtchenko and Rusakov, 2005; Zheng *et al*., 2008). The fluorophores were loaded into a patch pipette (1.5–2.0 MΩ) connected to a Picospritzer III (Parker Hannifin Precision Fluidics Division, Hollis, NH) and pressure applied in free medium (ACSF) or in hippocampal slices (∼50 μm below the slice surface). Each pressure application (∼13 PSI) lasted for 10 ms. The puff was triggered 200 ms after the beginning of the image acquisition. The two-photon laser scanning system (Scientifica, Uckfield, UK) was powered by a Ti:sapphire pulsed laser (Coherent, Bloomfield, CT) tuned to 810 nm and connected to an upright microscope with a 60 × 1.0 NA objective (Olympus Corporation). The green and red fluorescent signals were separated by a 565 nm dichroic mirror and filtered using FITC and TRITC filter sets (Olympus Corporation), respectively. We performed line scans (625 Hz) orthogonally to the pipette, at the focal plane of the tip, 5–10 μm away from it^32^. Ten consecutive line scans (8 s) were collected every 30 s using each pipette. The lines scans were averaged in Fiji (http://fiji.sc/Fiji) and analyzed in IgorPro 6.36 (Wavemetrics) with custom-made software (A.S.). We measured the light intensity profiles every 150 ms, before and after the puff. Each fluorescence profile was fitted with a Gaussian function (Levenberg-Marquardt algorithm, IgorPro 6.36) and the pre-puff fluorescence profile was routinely subtracted from the other recorded diffusion profiles as to avoid any bias from residual fluorescence between puffs. The linearity of diffusion of the fluorophores from a point source (the pipette) was verified in a 2 s time window after the puff and the diffusion coefficient (*D*_free_ or D*) was estimated from the linear regression of *γ*_*i*_^2^/4 versus time, where *γ* represents the width of the Gaussian profile at time *i*. For each fluorophore, the tortuosity (*λ*) was estimated as:


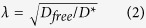


The hydration diameter (*d*_*H*_) was estimated as:


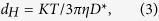


where *K* = Boltzmann’s constant (1.38·10^−23^ J/K), *T* = temperature in °K (295°K), *η* = viscosity of water (9.68·10^−4^ Pa·s) and D* = apparent diffusion coefficient of each fluorophore in control and TFLLR-treated slices.

### Electron microscopy and axial STEM tomography

Acute hippocampal slices processed for electron microscopy analysis were prepared as described for the electrophysiology experiments. Slices were microwave fixed for 13 s in 6% glutaraldehyde, 2% PFA, 2 mM CaCl_2_ in 0.1 N sodium cacodylate buffer^67^ and stored overnight at 4 °C. After 3 washes in 0.1 N cacodylate buffer, we cut samples from the middle part of CA1 *stratum radiatum*, ∼100 μm away from the pyramidal cell layer. These samples were treated with 1% osmium tetroxide for 1 hour on ice, *en bloc* mordanted with 0.25% uranyl acetate at 4 °C overnight, washed and dehydrated with a graded series of ethanol, and embedded in epoxy resins. Thin sections (70–90 nm) were counterstained with lead citrate and uranyl acetate and examined on a JEOL 1200 EX transmission electron microscope. Images were collected with a CCD digital camera system (XR-100, AMT). To visualize the arrangement of pre-synaptic terminals, post-synaptic terminals, and astrocytic processes, thick sections (∼1 μm) were cut from regions of CA1 *stratum radiatum* and electron tomograms were collected in a 300 kV electron microscope operated in the scanning transmission electron microscopy (STEM) mode, as described previously (Hohmann-Marriott *et al*., 2009; Sousa *et al*., 2011). A sample thickness of 1 μm – enabled by axial STEM tomography^68^,^69^ – provides sufficient sample depth to visualize features of interest in their entirety, such as synapses. In contrast to standard TEM tomography, conventional TEM tomography is limited to a specimen thickness of ∼400 nm and cannot be applied to such thick sections because the transmitted electrons undergo multiple inelastic scattering processes, resulting in images that are blurred by chromatic aberration of the objective lens. Axial STEM tomography is not affected by chromatic aberration because the objective lens that forms the electron probe is in front of the specimen. Recently, axial STEM tomography has been applied to image neuronal structures in retina^70^ and brain^71^. Dual-axis tilt series of selected sections were recorded using an FEI Tecnai TF30 TEM/STEM operating at 300 kV (1.5° tilt increment, tilt range from 55° to −55°, pixel size = 1.4 nm). Image registration, tomogram generation, tracing, surface area and volume measures were performed using IMOD 4.7 (http://bio3d.colorado.edu/imod/). The tomograms did not contain full astrocytes. However, in each tomogram, we identified astrocytic processes based on their shape and cytoplasmic structure^12^. Accordingly, each astrocytic process lacked synaptic vesicles and post-synaptic densities and did not give rise to pre- or post-synaptic terminals. The astrocytic processes contained glycogen granules, intermediate filament bundles and a clearer cytoplasm with respect to that of neurons. The astrocytic processes were traced for the entire thickness of the reconstructed volume (∼1 μm). We reconstructed all the nearest neighboring astrocytic processes that separated our synapse of interest from neighboring excitatory synapses. Typically, these astrocyte processes were located <600 nm away from the selected synapse, which is in agreement with available estimates of the mean nearest-neighbor distance between excitatory synapses in CA1 *stratum radiatum* (∼465 nm)^72^. We reconstructed a total of 25 synapses (Ctrl: n = 12; TFLLR: n = 13), randomly distributed within the axial STEM tomography blocks.

The reconstructed volumes were converted into object files and imported into the open-source software Blender 2.76 (https://www.blender.org/). Astrocyte-PSD distance analysis was performed in Blender using custom-made analysis software written in Python (M.F.R. and A.S.).

### 3D Monte Carlo reaction-diffusion simulations

We performed two sets of 3D Monte Carlo reaction-diffusion simulations using Blender 2.75 (Windows) or Blender 2.69 (Linux) and the CellBlender 1.0.1 add-on (http://mcell.org/). The geometrical parameters were based on the ones measured in the axial STEM tomography reconstructions of control sections and of sections treated with TFLLR (Table 1). In the first set of simulations, we generated simplified geometries of individual synaptic contacts and astrocytic processes (Fig. 4, Table 2). Each simulation was run within a 1 μm^3^ world. The pre-synaptic terminals (volume Ctrl: 0.079 μm^3^, TFLLR: 0.065 μm^3^; radius Ctrl: 0.27 μm, TFLLR: 0.24 μm) and post-synaptic terminals (volume Ctrl: 0.040 μm^3^, TFLLR: 0.029 μm^3^; radius Ctrl: 0.27 μm, TFLLR: 0.24 μm) were placed at the center of the world and separated from each other by a synaptic cleft of 20 nm height. The control synapse was surrounded by 2 astrocytic processes, each with a surface area of 0.69 μm^2^ (total astrocyte surface area: 1.38 μm^2^). The TFLLR synapse was surrounded by 4 astrocytic processes, each with a surface area of 0.54 μm^2^ (total astrocyte surface area: 2.18 μm^2^). The mean distance of the astrocytic processes from the center of the PSD was 116 nm in the control and 190 nm in the TFLLR simulation. Each astrocytic process was covered by glutamate transporters (10,800 μm^−2^)^73^. The geometrical parameters for the second set of simulations is included in Table 3. The PSD expressed GluA and GluN receptors (200 and 300 μm^−2^, respectively)^45^,^46^,^74^. GluA and GluN receptors were also expressed, at lower density, in the extra-synaptic regions (40 and 60 μm^−2^, respectively)^29^,^33^. The GluN and GluA kinetic rates were set in accordance to^75^ and^76^, respectively. The glutamate transporters were modeled using the simplified kinetic scheme shown in Fig. 4H^77^. The time constant for glutamate translocation across the membrane was set to *k*_*trans*_ = 2,000s^−1^ 78; the transporter reorientation rate was set to *k*_*reorient*_ = 50 s^−1 ^77. The scheme included a rapid binding and unbinding rates (*k*_*on*_ and *k*_*off*_ respectively). The binding rate was set to *k*_*on*_ = 6e6 M^−1^ s^−1 ^78. The unbinding rate was derived from the equation of the apparent steady state affinity for glutamate:


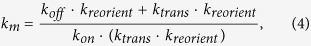


in which *k*_*m*_ = 13 μM^18^. The derived value for the unbinding rate was *k*_*off*_ = 601. All kinetic rates were adjusted for Q_10_=3, to approximate the receptor and transporter kinetics at physiological temperature (35 °C). At the beginning of each simulation, 2,000 glutamate molecules were released from a point source placed in the center of the synaptic cleft, close to the pre-synaptic terminal^79^. In previous work, we showed that the surface density of glutamate transporters (10,800 μm^−2^) is orders of magnitude larger than the number of glutamate molecules released from an individual synaptic contact. Varying the number of released glutamate molecules over a wide range from 1,000 to 20,000 in 3D reaction-diffusion simulations similar to those used in the present work did not show any appreciable effect on the time course of glutamate clearance from astrocytes (Supp. Fig. 9 in ref. 29). For this reason, we released 2,000 glutamate molecules, a number that is in close agreement with currently available estimates of the quantal size of glutamatergic synaptic vesicles^79^. Glutamate diffused within the synaptic cleft with a diffusion coefficient D* = 3.3e-6 cm^2^/s^80^. The apparent diffusion coefficient was set to 1.41e-6 cm^2^/s outside of the synaptic cleft, to account for the tortuosity of the hippocampal neuropil (*λ*), which describes the hindrance to free diffusion experienced by glutamate as it diffuses in the hippocampal neuropil (*λ* = 1.6)^81^. The tortuosity factor *λ* can be described as:


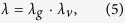


where *λ*_*g*_ and *λ*_*v*_ represent the geometrical and viscous components of *λ*. Previous work estimated *λ*_*g*_ = 1.48 29 and therefore *λ*_*v*_ = 1.38. In the simulations ran with simplified geometries, only 18% of the simulation volume is occupied by the synaptic elements and astrocytic processes and therefore 
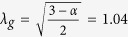
. Therefore, in these simulations, the tortuosity is:


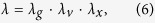


where *λ*_*x*_ is a correction factor to obtain *λ*=1.6. In our case, *λ*_*x*_ = 1.11. The tortuosity can also be expressed as a function of the free and apparent diffusion coefficients (*D*_*free*_ and D*, respectively), accordingly to the formula:


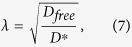


from which we obtain:


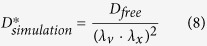


(i.e. 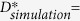
1.41e-6 cm^2^/s). Each simulation consisted of 5,000 iterations with a time step *Δt* = 10 μs (therefore spanning a 50 ms time window), and was run for 300 seeds. We averaged the results of all 300 seeds using custom-made software written in Python 3.5 (https://www.python.org/). All surfaces for the geometries included in the simulation scene were reflective for glutamate. The glutamate concentration and the number of glutamate transporter binding, unbinding and translocation reactions were monitored at every time step, in the synaptic cleft and in the extracellular space.

### Analytical simulations

Analytical simulations of GluA-EPSCs were performed in ChanneLab (Synaptosoft Inc., Decatur, GA), with a time interval=0.01 ms, V_hold_=−70 mV, RK4 integration and step size=5 μs (Fig. 6H–J). All kinetic rates were taken from^42^. Data were imported in IgorPro 6.36 to obtain a contour plot (Fig. 6H).

### Statistical analysis

Data are presented as mean ± S.E.M, unless otherwise stated. Statistical significance was determined by Student’s paired or unpaired t-test, as appropriate (IgorPro 6.36). Differences were considered significant at p < 0.05 (*p < 0.05; **p < 0.01; ***p < 0.001).

## Additional Information

**How to cite this article:** Sweeney, A. M. *et al*. PAR1 activation induces rapid changes in glutamate uptake and astrocyte morphology. *Sci. Rep.* **7**, 43606; doi: 10.1038/srep43606 (2017).

**Publisher's note:** Springer Nature remains neutral with regard to jurisdictional claims in published maps and institutional affiliations.

## Supplementary Material

Supplementary Information

## Figures and Tables

**Table 1:** Geometrical properties of excitatory synapses reconstructed with axial STEM tomography.

	Ctrl	TFLLR
PSD surface area	0.10 ± 0.11 μm^2^ (n = 12)	0.08 ± 0.05 μm^2^ (n = 13)
Pre-synaptic volume	0.08 ± 0.08 μm^3^ (n = 12)	0.06 ± 0.03 μm^3^ (n = 13)
Post-synaptic volume	0.04 ± 0.04 μm^3^ (n = 12)	0.03 ± 0.02 μm^3^ (n = 13)
Mean astrocytic process volume	0.03 ± 0.02 μm^3^ (n = 27)	0.02 ± 0.01 μm^3^ (n = 55) *p = 0.025
Mean astrocytic process SA	0.80 ± 0.45 μm^2^ (n = 27)	0.57 ± 0.22 μm^2^ (n = 55) *p = 0.039
Mean number of astrocytic processes at each synapse	2.25 ± 1.54 (n = 12)	4.23 ± 2.38 (n = 13)
Mean PSD-astrocytic process distance at each synapse	116.5 ± 55.4 nm (n = 12)	190.9 ± 74.6 nm (n = 13)
Mean astrocytic process volume of each synapse	0.03 ± 0.02 μm^3^ (n = 11)	0.02 ± 0.01 μm^3^ (n = 13) *p = 0.031
Mean astrocytic process SA at each synapse	0.91 ± 0.43 μm^2^ (n = 11)	0.58 ± 0.15 μm^2^ (n = 13) *p = 0.029
Total astrocytic process volume at each synapse	0.06 ± 0.03 μm^3^ (n = 12)	0.07 ± 0.05 μm^3^ (n = 13)
Total astrocytic process SA at each synapse	1.79 ± 1.07 μm^2^ (n = 12)	2.52 ± 1.63 μm^2^ (n = 13)

**Table 2:** Geometrical properties of excitatory synapses used for 3D Monte Carlo reaction-diffusion simulations (Fig. 4).

	Ctrl	TFLLR
PSD surface area	0.10 μm^2^	0.10 μm^2^
Pre-synaptic volume	0.07 μm^3^	0.06 μm^3^
Post-synaptic volume	0.04 μm^3^	0.03 μm^3^
Mean astrocytic process volume	0.05 μm^3^	0.10 μm^3^
Mean astrocytic process SA	0.89 μm^2^	0.60 μm^2^
Number of astrocytic processes	2	4
PSD- Astrocytic process distance	116 nm	190 nm
Total astrocytic process volume at each synapse	0.10 μm^3^	0.10 μm^3^
Total astrocytic process SA at each synapse	1.78 μm^2^	2.40 μm^2^

**Table 3:** Geometrical properties of reconstructed excitatory synapses used for 3D Monte Carlo reaction-diffusion simulations (Fig. 5).

	Ctrl	TFLLR
PSD surface area	0.14 μm^2^	0.04 μm^2^
Pre-synaptic volume	0.15 μm^3^	0.05 μm^3^
Post-synaptic volume	0.02 μm^3^	0.01 μm^3^
Mean astrocytic process volume	0.01 μm^3^	0.02 μm^3^
Mean astrocytic process SA	0.53 μm^2^	0.75 μm^2^
Number of astrocytic processes	2	4
PSD- Astrocytic process distance	85 nm	184 nm
Total astrocytic process volume at each synapse	0.03 μm^3^	0.07 μm^3^
Total astrocytic process SA at each synapse	1.07 μm^2^	2.99 μm^2^
