# Supplementary material for: PAR1 activation induces rapid changes in glutamate uptake and astrocyte morphology
Source: Sci Rep. 2017 Mar 3;7:43606. doi: 10.1038/srep43606 (PMC5335386; doi:10.1038/srep43606)
Supplement: Supplementary Information [file srep43606-s1.pdf]

TITLE PAGE

TITLE

PAR1 activation induces rapid changes in glutamate uptake and astrocyte morphology

AUTHORS

Amanda M. Sweeney*†^, Kelsey E. Fleming*^, John P. McCauley*, Marvin F. Rodriguez*ǀ, Elliot T. Martin*, Alioscka A. Sousa#‡, Richard D. Leapman# and Annalisa Scimemi*

AFFILIATIONS

* SUNY Albany, Dept. Biology, 1400 Washington Avenue, Albany (NY) 12222, USA

ǀ SUNY Oneonta, Dept. Computer Science, 108 Ravine Parkway, Oneonta (NY) 13820, USA

\# National Institute of Biomedical Imaging and Bioengineering, National Institutes of Health, 9000 Rockville Pike, Bethesda (MD) 20852, USA

^ These authors contributed equally to this work

CURRENT ADDRESS

† University of Rochester Medical Center, 77 Ridgeland Road, Rochester (NY) 14623, USA

ǀ SUNY Oneonta, IT Enterprise Application Services, 108 Ravine Parkway, Oneonta (NY) 13820, USA

‡ Univ. Federal de São Paulo, Dept. Biochemistry, Rua Três de Maio nº 100, São Paulo 04044, Brazil

CORRESPONDING AUTHOR

Dr. Annalisa Scimemi, PhD

SUNY Albany, Dept. Biology, 1400 Washington Avenue, Albany (NY) 12222, USA

Tel.: +1-518-442-4367

Fax: +1-518-442-4767

Email: scimemia@gmail.com or ascimemi@albany.edu

WORD COUNTS AND LIMITS

Abstract:	198 words	(limit: 200)
Main text:	4,485 words	(limit 4,500)
Figures:	8	(limit: 8)
Figure legends:	350 words	(all within this limit)

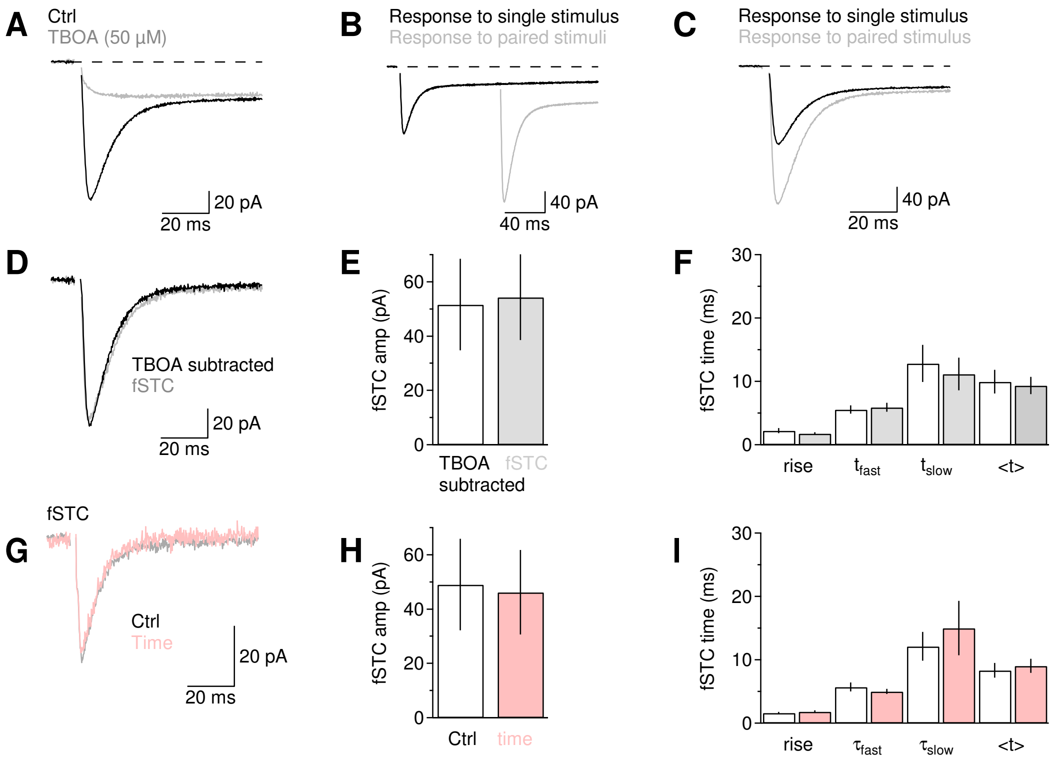

Supplementary Figure 1

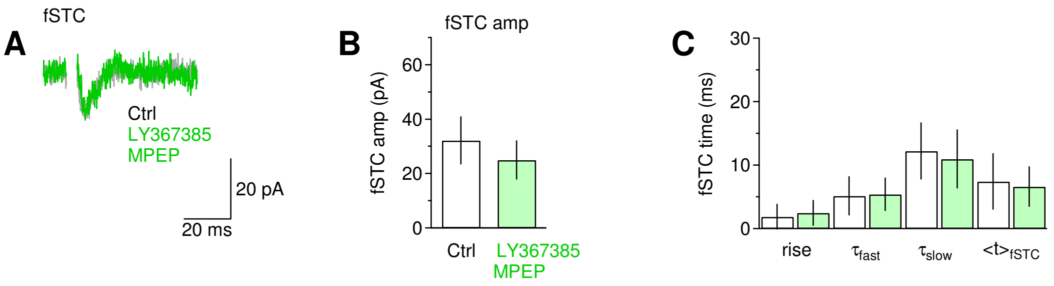

Supplementary Figure 2

Supplementary Figure 1. Comparison of pharmacologically-isolated and paired-pulse subtracted STCs. **(A)** Example of STCs recorded in control solution (*black*) and in the additional presence of TBOA (50 µM; *gray*). TBOA blocked the fast rising and fast decaying component of the recorded current and did not block the slow-rising and sustained K^+ current. **(B)** Astrocyte current responses, evoked by delivering single (*black*) and paired stimuli to Schaffer collaterals. **(C)** Overlay of currents evoked by the first (*black*) and second (*gray*) of two paired stimuli. The response to the second stimulus is obtained by subtracting the single from the paired response. The response to the paired stimulus is shifted in time for comparison with the single response. Transporter-mediated currents facilitate more than the potassium current. The facilitated component of the STC (fSTC) is obtained by subtracting the single from the paired response. **(D)** Overlay of transporter currents isolated pharmacologically using TBOA (*black*) and analytically using the subtraction method (fSTC, *gray*). **(E)** Summary graph: TBOA subtracted currents and the fSTCs have similar amplitude (amp $STC_{TBOA\ sub}$ 51.6±16.8 pA, fSTC 54.3±15.8 pA, p=0.54). **(F)** Same astrocytes as in (E). Summary graph: TBOA subtracted currents and fSTCs have similar kinetics (20-80% rise $STC_{TBOA\ sub}$ 2.2±0.4 ms, fSTC 1.8±0.2 ms p=0.36; τ_{fast} $STC_{TBOA\ sub}$ 5.5±0.7 ms, fSTC 5.9±0.7 ms p=0.70; τ_{slow} $STC_{TBOA\ sub}$ 12.8±0.7 ms, fSTC 11.2±2.6 ms p=0.57; <t>$_{TBOA\ sub}$ 10.0±1.9 ms, fSTC 9.4±1.3 ms (n=4) p=0.84). **(G)** Overlay of fSTCs recorded 10 min (*gray*) and 30 min (*pink*) after establishing the whole-cell patch clamp configuration. **(H)** Summary graph: the fSTC amplitude does not change over the time course of our astrocyte recordings (amp $fSTC_{Ctrl}$ 49.0±16.8 pA, $fSTC_{time}$ 46.2±15.6 pA p=0.26). **(I)** Same astrocytes as in (H). Summary graph: the kinetics of the fSTC remain unaltered over the time course of our recordings (~30 min; (20-80% rise $fSTC_{Ctrl}$ 1.6±0.1 ms, $fSTC_{time}$ 1.8±0.2 ms p=0.09; τ_{fast} $fSTC_{Ctrl}$ 5.7±0.7 ms, $fSTC_{time}$ 5.0±0.4 ms p=0.42; τ_{slow} $fSTC_{Ctrl}$ 12.1±2.3 ms, $fSTC_{time}$ 15.0±4.3 ms p=0.32; <t> $fSTC_{Ctrl}$ 8.3±1.2 ms, $fSTC_{time}$ 9.0±1.1 ms (n=4) p=0.14). All recordings in panels A-D,G represent the average of 20 consecutive traces.

Supplementary Figure 2. Effect of mGluRI antagonists on fSTCs. **(A)** Overlay of fSTCs recorded in control conditions (*gray*) and in the presence of the mGluRI antagonists LY367385 (50 µM) and MPEP (10 µM). **(B)** Summary graph: the fSTC amplitude is similar in control conditions and in the presence of mGluRI antagonists (amp $fSTC_{Ctrl}$ 32.2±8.8 pA, $fSTC_{mGluRI}$ 24.9±7.2 pA (n=9) p=0.09). **(C)** The kinetics of fSTCs are not altered by the mGluRI antagonists LY367385 (50 µM) and MPEP (10 µM; 20-80% rise $fSTC_{Ctrl}$ 1.8±0.3 ms, $fSTC_{mGluRI}$ 2.5±0.5 ms p=0.07; τ_{fast} $fSTC_{Ctrl}$ 5.2±0.9 ms, $fSTC_{mGluRI}$ 5.4±1.4 ms p=0.88; τ_{slow} $fSTC_{Ctrl}$ 12.7±2.7 ms, $fSTC_{mGluRI}$ 12.0±1.7 ms p=0.82; <t> $fSTC_{Ctrl}$ 7.4±4.4 ms, $fSTC_{time}$ 6.6±3.2 ms (n=9) p=0.55).
